# Combining CD38 antibody with CD47 blockade is a promising strategy for treating hematologic malignancies expressing CD38

**DOI:** 10.3389/fimmu.2024.1398508

**Published:** 2024-06-25

**Authors:** Song Li, Dianze Chen, Yanan Yang, Huiqin Guo, Dandan Liu, Nana Sun, Xing Bai, Kaili Wang, Tengfei Li, Guanghui Li, Chunmei Yang, Wei Zhang, Li Zhang, Gui Zhao, Liang Peng, Sijin Liu, Xiaoping Tu, Ruliang Zhang, Wenzhi Tian

**Affiliations:** ^1^ Department of R&D, ImmuneOnco Biopharmaceuticals (Shanghai) Inc., Shanghai, China; ^2^ Department of CMC, ImmuneOnco Biopharmaceuticals (Shanghai) Inc., Shanghai, China

**Keywords:** CD38, cd47, CD38/CD47 bispecific antibody, hematologic malignancies, Fc-mediated effector function, Apoptosis

## Abstract

**Background:**

CD38 and CD47 are expressed in many hematologic malignancies, including multiple myeloma (MM), B-cell non-Hodgkin lymphoma (NHL), B-cell acute lymphoblastic leukemia (ALL), and B-cell chronic lymphocytic leukemia (CLL). Here, we evaluated the antitumor activities of CD38/CD47 bispecific antibodies (BsAbs).

**Methods:**

Five suitable anti-CD38 antibodies for co-targeting CD47 and CD38 BsAb were developed using a 2 + 2 “mAb-trap” platform. The activity characteristics of the CD38/CD47 BsAbs were evaluated using *in vitro* and *in vivo* systems.

**Results:**

Using hybridoma screening technology, we obtained nine suitable anti-CD38 antibodies. All anti-CD38 antibodies bind to CD38^+^ tumor cells and kill tumor cells via antibody-dependent cellular cytotoxicity (ADCC) and antibody-dependent cellular phagocytosis (ADCP). Five anti-CD38 antibodies (4A8, 12C10, 26B4, 35G5, and 65A7) were selected for designing CD38/CD47 BsAbs (IMM5605) using a “mAb-trap” platform. BsAbs had higher affinity and binding activity to the CD38 target than those to the CD47 target, decreasing the potential on-target potential and off-tumor effects. The CD38/CD47 BsAbs did not bind to RBCs and did not induce RBC agglutination; thus, BsAbs had much lower blood toxicity. The CD38/CD47 BsAbs had a greater ability to block the CD47/SIRPα signal in CD38^+^/CD47^+^ tumor cells than IMM01 (SIRPα Fc fusion protein). Through Fc domain engineering, CD38/CD47 BsAbs were shown to kill tumors more effectively by inducing ADCC and ADCP. IMM5605–26B4 had the strongest inhibitory effect on cellular CD38 enzymatic activity. IMM5605–12C10 had the strongest ability to directly induce the apoptosis of tumor cells. The anti-CD38 antibody 26B4 combined with the SIRPα-Fc fusion proteins showed strong antitumor effects, which were better than any of the mono-therapeutic agents used alone in the NCI-H929 cell xenograft model. The CD38/CD47 BsAbs exhibited strong antitumor effects; specifically, IMM5605–12C10 efficiently eradicated all established tumors in all mice.

**Conclusion:**

A panel of BsAbs targeting CD38 and CD47 developed based on the “mAb-tarp” platform showed potent tumor-killing ability *in vitro* and *in vivo*. As BsAbs had lower affinity for binding to CD47, higher affinity for binding to CD38, no affinity for binding to RBCs, and did not induce RBC agglutination, we concluded that CD38/CD47 BsAbs are safe and have a satisfactory tolerability profile.

## 1 Introduction

The CD38 protein is a 45 kDa type II transmembrane glycoprotein comprising a long extracellular domain (258 aa) and a short N-terminal cytoplasmic tail (21 aa). It is a suitable therapeutic target for many human hematologic malignancies. CD38 occurs in biological fluids in a 39 kDa soluble form through internalization or shedding (1). Under physiological conditions, CD38 expression in lymphoid cells, myeloid cells, and some non-hematopoietic tissues is relatively low (2). However, normal plasma and multiple myeloma (MM) cells exhibit increased CD38 levels, making CD38 a suitable target for developing therapeutic antibodies against MM (3). CD38 is also expressed in regulatory T (Tregs) and B (Bregs) cells, as well as in myeloid-derived suppressor cells (MDSCs). They are expressed at high levels on the surface, which is linked with compromised immune surveillance in malignancies (4–6). CD38 serves as a cell surface receptor for CD31 and essentially controls migration, signaling events, and receptor-mediated adhesion (7). Furthermore, CD38 has an ectoenzymatic role and is involved in the production of nucleotide metabolites, which regulate intracellular calcium stores (8, 9). Some studies have suggested the involvement of CD38, which has immunosuppressive activity, in adenosine generation (10). CD38 antibodies indicate pleiotropic mechanisms of action, such as direct apoptotic function, Fc-dependent immune-effector mechanisms, immunomodulation by CD38^+^ immune-suppressor cell removal, and suppression of ectoenzymatic activities. Many CD38 antibodies are currently in the pipeline at various phases of clinical assessments. In 2015, daratumumab was discovered as the first monoclonal antibody that targets CD38 and was approved for treating MM for various indications, combined with standards of care and used as monotherapy (11). Isatuximab is also a CD38-targeting monoclonal antibody that was authorized by the United States Food and Drug Administration for the treatment of relapsed MM. It was given in combination with dexamethasone (Isa-Kd) and carfilzomib, as well as with dexamethasone (Isa-Pd) and pomalidomide (12).

The CD47 protein is a 50 kDa type I transmembrane glycoprotein that is ubiquitously expressed in healthy cells but is overexpressed in various tumor cells. It has a C-terminal cytoplasmic tail, five transmembrane helices, and one N-terminal extracellular Ig-like domain. It belongs to the immunoglobulin family that acts as a “do not eat me” signal (13). The CD47 ligands include thrombospondin-1 (TSP-1), signal-regulatory protein α (SIRPα), and integrins (α2β1 and αvβ3) (13). Upon binding of CD47 to SIRPα expressed on the surface of dendritic cells and macrophages, immunoreceptor tyrosine-based inhibitory motif (ITIM) can trigger a cascade to inhibit phagocytosis (14). CD47 is strongly expressed in various solid tumors and hematologic malignancies (15, 16). Additionally, a high level of CD47 expression was found to be linked to substandard patient prognosis in different types of cancer (17–19). Cancer cells upregulate the expression of CD47 to evade immune surveillance and subsequent destruction. To prevent the evasion of immunosurveillance via CD47-SIRPα signaling, several immunotherapeutics are under clinical evaluation (clinicaltrials.gov; e.g., NCT03013218, NCT02890368, NCT02953509, and NCT02367196).

In some trials, the effect of combining CD38 antibodies with CD47 blockade has been investigated. I-Mab Biopharma tests the combination of lemzoparlimab (anti-CD47 IgG4 antibody) and felzartamab (anti-CD38 IgG1 antibody) for treating relapsed/refractory (R/R) MM patients (NCT04895410). The results of a preclinical study showed that administering a combination of felzartamab and lemzoparlimab increased *in vivo* antitumor efficacy and *in vitro* ADCP in CD38-low and CD47-high R/R MM cells, which were resistant to daratumumab or felzartamab single-drug treatment (20). Bispecific antibodies are a new and effective treatment option for cancer patients. Several researchers have investigated the efficacy of the CD38 and CD47 bispecific antibody (BsAb) ISB 1442 and assessed its pharmacokinetics (PK), tolerability, safety, efficacy, and pharmacodynamics (PD) in R/RMM patients (NCT05427812). Preclinically, ISB 1442 exhibited increased killing efficiency compared with daratumumab when used against tumors expressing high and low levels of CD38. Because of its reduced binding affinity for CD47, ISB 1442 only interacts with CD47 efficiently after it binds to CD38 (avidity-induced binding). This characteristic of ISB 1442 reduces the potential for off-tumor and on-target activity, and it does not deplete RBCs *in vitro* unlike magrolimab (21). CD38 and CD47 are also expressed in other hematologic cancers, such as T-cell acute lymphoblastic leukemia (T-ALL) and acute myeloid leukemia (AML). Dual CD47 and CD38 targeting enhances antibody-mediated phagocytosis in T-ALL cell lines and R/R T-ALL PDX samples *in vitro* (22). Moreover, compared with anti-CD47 (5F9) and daratumumab, ISB 1442 also mediates increased phagocytosis of T-ALL and AML tumor cells in cell lines with reduced CD38 and CD47 expression (23).

In this study, we reported a novel approach for treating CD38^+^ malignancies by co-targeting both CD47 and CD38 bispecific antibodies using a “mAb-trap” platform. Because of its distinctive design and diverse modes of action, the CD38/CD47 BsAb has the potential to augment antitumor efficacy in individuals with CD38^+^ malignancies in comparison to anti-CD38 mAbs by effectively addressing primary and acquired tumor escape mechanisms of resistance. Our findings suggested that dual targeting of CD38 and CD47 is a promising strategy for treating CD38^+^ malignancies, especially in patients with relapsed/refractory diseases with a poor prognosis.

## 2 Materials and methods

### 2.1 Cell culture

The Raji, SP2/0, Jurkat, Reh, and Daudi cell lines were acquired from the Cell Bank of the Chinese Academy of Sciences. The NCI-H929 and NK92MI cell lines were obtained from the American Type Culture Collection (ATCC). L-1236 and MOLM-13 cell lines were purchased from Shanghai Qiansi Biotechnology Co., Ltd., and Nanjing Cobioer Biosciences Co., Ltd., respectively. The modification of CHO-CD38 and FcgRIIIA (158 V) target-stimulated NK (FcR-TANK™) cells were carried out in our laboratory. Cells in all cell lines were harvested after they reached the logarithmic growth phase. Jurkat, SP2/0, L-1236, Daudi, MOLM-13, Reh, Raji, and NCI-H929 cells were incubated at 37°C in 5% CO_2_. RPMI-1640 media (Gibco, Cat#11875093) was utilized for the cultivation of all the cells. The media were supplemented with 1% penicillin–streptomycin (Gibco, Cat# 15140122) and 10% fetal bovine serum (Gibco, Cat# 10091148). CHO-CD38 cells were cultured in EX-CELL^®^ 302 serum-free medium (Sigma, Cat# 24326C) supplemented with 1% penicillin–streptomycin. Serum-negative TANK media (Immuneonco, Cat# CT001–1) was utilized for cultivating FcR-TANK cells.

### 2.2 Development of anti-CD38 antibodies

After BALB/c mice were immunized with CHO cells overexpressing hCD38 and the human CD38 full-length extracellular domain (43–300) fused to mIgG1-Fc, the conventional hybridoma method was used to screen for anti-human CD38 antibodies. Positive fusions were assessed for specific interactions with CHO-CD38, but not with CHO, by fusing immunized animal splenocytes with Sp2/0 myeloma cells. Based on the epitope and sequence homology, we selected nine anti-CD38 antibodies for detailed investigation and development of CD38/CD47 BsAbs.

### 2.3 Protein expression, purification, and characterization

TransFx-C CHO transient transfection media (HyClone, Cat# SH30942.02) was utilized for the cultivation of CHO-S cells (Gibco, Cat# A29127). By transient transfection, the expression vectors expressing the heavy and light chains of antibodies were cotransfected using a polyethylenimine transfection reagent (Polysciences, Cat# 24765). The supernatant of the cotransfected cells was collected after 8–10 days and added to Protein A Sepharose columns (Bestchrom, Cat# AA0273). Subsequently, wash buffer [NaCl (140 mM) + phosphate buffer (PB; 20 mM); pH = 7.4 ± 0.1] was added to the columns, and elution buffer [NaAc (25 mM) + NaCl (100 mM); pH = 3.5 ± 0.1] was used to elute the antibodies. The pH of the collected fraction was adjusted to 5.2 ± 0.2 with 2 M Tris. Finally, size-exclusion high-performance liquid chromatography (SEC-HPLC) was carried out to determine the purity of the acquired antibodies.

### 2.4 Biolayer interferometry technology analysis of affinity, epitope binning, and dual-target binding

#### 2.4.1 Affinity determination assay

For the BLI assay, the Gator™ Label-Free Bioanalysis instrument (Gator Bio) was utilized. Samples were measured by two probes (sample and reference). For the assay setup, the anti-human IgG probes (Gator Bio, Cat#20–5036) were pre-incubated for 300 s in Q buffer (10 mM PBS +0.02% tween + 0.2% BSA; pH = 7.4). The measurements using a sample probe started at a 20 s baseline in a Q buffer. Then, probe loading was performed for 60 s with 10 µg/mL antibody, probe washing was performed for 20 s in Q buffer, probe incubation was performed for 60 s in CD38 antigen (Kactus Biosystems, Cat# CD3-HM138) or CD47 antigen (Sino Biological, Cat# 12283-HCCH) wells (association step), and probe incubation was performed for 60 s in Q buffer (dissociation step). The reference probe measurements and probe measurement methods were identical, but no antibody was loaded, and instead, the reference probe was incubated in Q buffer. The curves were processed using Gator software with a 1:1 fit after subtracting the background.

#### 2.4.2 Epitope binning assay

We used the BLI-based in-tandem orientation method to analyze the epitope binning of anti-CD38 antibodies. The assessment using the anti-His probe was initiated with a 20 s baseline measurement in Q buffer. This was followed by probe loading with 10 µg/mL His-Tag CD38 antigen for 120 s, 20 s of probe rinsing with Q buffer, 120 s of incubation in a 10 µg/mL Ab1 well, and subsequent rinsing in a 10 µg/mL Ab2 well for 60 s.

#### 2.4.3 Dual-target binding determination assay

The BLI-based tandem method was used to evaluate the ability of CD38/CD47 BsAbs to bind dual targets. The measurement with an anti-human IgG probe was initiated with a 20 s baseline measurement in Q buffer. This was followed by probe loading with 10 µg/mL CD38/CD47 BsAbs for 120 s, 20 s of probe rinsing with Q buffer, 60 s of probe incubation in a 10 µg/mL Ag1 well, and 30 s of probe incubation in a 10 µg/mL Ag2 well containing 10 µg/mL Ag1.

### 2.5 Binding to CD38+ tumor cells, RBCs, and platelets

The antibodies binding to CD38+ tumor cells (including Raji, Daudi, NCI-H929, L-1236, MOLM-13, and Reh), RBCs, and platelets were assessed via flow cytometry. hIgG1-Fc (in-house) was used as an isotype reference. The samples were incubated at 4°C in tumor cells at multiple serial dilutions for 45 min. Then, PBS + 0.5% BSA (Sangon Biotech; Cat#A500023–0100) was added to remove the free antibodies by centrifugation. Subsequently, the samples were treated with FITC-linked anti-human IgG Fc secondary antibodies (500-fold dilution; Sigma, Cat# F9512) at 4°C in the dark for 45 min. After washing, the cells’ FITC fluorescence signals were assessed by flow cytometry analysis (Luminex, Guava^®^ easyCyte™ 8HT Base System). The acquired data were analyzed using guavaSoft_33_x64 software.

### 2.6 Blocking the CD47/SIRPα interaction assay

Similar to the above binding assay, after Jurkat or Reh cells were pretreated with various concentrations of the CD38/CD47 BsAb for 30 min, in this mixture, 1 µg/mL SIRPα-mFc (in-house) was added at a 1:1 (v/v) ratio for 45 min. The cells were then washed with PBS supplemented with 0.5% BSA, the secondary antibody PE-conjugated anti-mouse IgG (500-fold dilution; Biolegend, Cat# 405307) was added, and the cell PE fluorescence signal was detected via flow cytometry analysis.

### 2.7 CD38 enzyme activity inhibition assay

Using PBS, the antibodies were diluted to 10 µg/mL, and CD38^+^ tumor cells were resuspended to a density of 1 × 10^6^ cells/mL. The antibodies and cells were mixed at a 1:1 ratio (by volume) and added to a 96 well flat-bottom plate (BeyoGold, Cat#FCP968) containing 100 µL of the mixture in each well. For 15 min, the plates were pre-incubated in the dark at ambient temperature. The nicotinamide guanine dinucleotide (NGD) substrate solution (Sigma Aldrich, Cat# N5131) was diluted to 120 µM, and 50 µL of this solution was added to each well containing the mixture. Then, for 2 h, the plates were preincubated in the dark at ambient temperature. The production of cGDPR was quantified by reading fluorescence signals at an excitation wavelength of 300 nm (EX300) and an emission wavelength of 410 nm (EM400) using a multifunctional enzyme-labeling instrument (Molecular Devices, SpectraMax M3). The percent inhibition was calculated as follows: inhibition % = [(1-(Antibodies-blank)/(positive-blank))×100]%. The antibody group contained 50 µL of cell + 50 µL of antibody + 50 µL of NGD, the blank group contained 50 µL of cell + 100 µL of PBS, and the positive group contained 50 µL of cell + 50 µL of PBS + 50 µL of NGD.

### 2.8 Tumor cell apoptosis induction assay

The incubation of the target cells was carried out at 37°C in various concentrations of antibodies in 5% CO_2_ for 4 h before staining with propidium iodide (PI) solution (Sigma, Cat# P4170). The cells were collected by flow cytometry, and PI-positive cells were subsequently assessed.

### 2.9 *In vitro* Fc-mediated effector function (ADCC/ADCP/CDC) assay

#### 2.9.1 ADCC assay

The target cells were stained with carboxyfluorescein succinimidyl ester (CSFE; 200 nM; Sigma, Cat# 21888) and incubated with various concentrations of antibodies for 30 min. Then, at 37°C and a 1:2 E/T ratio, FcgRIIIA (158 V) target-activated NK (FcR-TANK™) cells (in-house) were added to the wells for 4 h in 5% CO_2_. The cells were dyed with PI solution, collected with a flow cytometer, and assessed for the percentage of PI-positive cells. The ADCC intensity was measured using the following formula: Lysis% = ((E+T+Ab) % PI-positive cell – (E+T) % PI-positive cell)/(100 – T % PI-positive cell) × 100%.

#### 2.9.2 ADCP assay

The THP-1 cells were collected and washed with RPMI-1640 media comprising 1% penicillin–streptomycin and 10% fetal bovine serum. Then, 200 ng/mL PMA (Sigma, Cat# P-050) and 100 µL of THP-1 cells (4 × 10^5^ cells/mL) were incubated for 48 h in a flat 96 well plate at 37°C and 5% CO_2_. Then, the target cells were counted, harvested, and labeled by incubation in the dark with 200 nM CSFE for 30 min at 37°C. The cells were then rinsed twice with complete culture media and propagated at a density of 1 × 10^5^ cells/well (50 µL, 2 × 10^6^/mL). The cells were treated with serially diluted antibodies at 50 µL/well for 2 h (Effector: Target = 2:5) at 37°C in an incubator with 5% CO_2_. The free target cells were removed by washing the plates with PBS. Then, the samples were resuspended in PBS, and the phagocytic index was assessed by flow cytometry and defined as the percentage of macrophages that phagocytosed the target cells.

#### 2.9.3 CDC assay

Different concentrations of antibodies were utilized for treating the target cells at 37°C with normal human serum complement (Quidel, Cat# A113) and 5% CO_2_ for 4 h. Then, PI staining was carried out, and flow cytometry analysis was carried out to collect the cells. The percentage of PI-positive cells was defined, and the CDC intensity was assessed by the following formula: Lysis % = Experimental Sample Lysis %- No Antibody Lysis %.

### 2.10 *In vivo* xenograft mouse model

The *in vivo* efficacy of CD38/CD47 BsAbs and the anti-CD38 antibody combined with the SIRPα Fc fusion protein was evaluated in NCI-H929 xenograft models. Further, 200 µL of 5 × 10^6^ cells were resuspended in cold PBS/Matrigel (v/v = 1:1) and administered on the right side of the CB17-SCID mouse back near the axilla. After the tumors reached an average volume of 120 mm^3^, the mice were randomly categorized into treatment groups based on tumor size and mouse weight. Drugs were injected intraperitoneally in all the experiments. The body weights and tumor volumes of the mice were determined twice per week, and when the tumor size reached 3,000 mm^3^, the mice were euthanized.

### 2.11 Statistical analysis

GraphPad Prism 8.0 (GraphPad Software, Inc.) was used for all the statistical analyses. The differences between three or more groups were elucidated by one-way ANOVA with Holm–Sidak correction, whereas the intergroup differences were elucidated by Student’s t-tests. p ≤ 0.05 indicated statistically significant differences among and between groups. In the figures, asterisks denote statistical significance (*p < 0.05; **p < 0.01; ***p < 0.001; ****p < 0.0001).

## 3 Results

In this study, we evaluated the ability of anti-CD38 antibodies to kill tumor cells via ADCC, CDC, ADCP, and apoptosis induction *in vitro* against a panel of hematologic cancer cell lines, including the MM cell line NCI-H929, and other cell lines derived from various hematologic malignancies, including non-Hodgkin’s lymphoma (Daudi and Raji), Hodgkin’s lymphoma (L-1236), acute lymphocytic leukemia (Reh), and acute myeloid leukemia (MOLM-13). Based on *in vitro* activity data, we selected five anti-CD38 antibodies for the development of CD38/CD47 BsAbs using a 2 + 2 “mAb-trap” platform. These CD38/CD47 BsAbs comprised a high-affinity CD38 targeting arm with a low-affinity CD47 blocking arm on an engineered human IgG1 Fc; this structure enhanced Fc-dependent effector functions, including ADCC, ADCP, and CDC. These CD38/CD47 BsAbs could selectively block the interaction between CD47 and SIRPα on CD38^+^/CD47^+^ tumor cells and induce tumor cell death *in vitro* and *in vivo*. We also used the backbone of daratumumab and isatuximab antibodies to design CD38/CD47 BsAbs as controls.

### 3.1 Anti-CD38 antibodies had rich epitopes and high sequence diversity

Using mouse hybridoma screening technology, we obtained nine ideal anti-CD38 antibodies that specifically target CD38. We analyzed epitopes using the BLI-based in-tandem orientation method, sequenced cDNA using the SMARTer^®^ RACE 5’/3’ Kit (Taraka, Cat#634858), and assessed sequence homology using DNAMAN software. The epitope binning technique is used to cluster different antibodies based on their recognized epitopes [epitopes are the specific regions on the antigen (Ag) recognized by the antibody (Ab)]. The results showed that the epitopes of the nine anti-CD38 antibodies were clustered into four categories: 35G5, 26B4, and 25B5 were in one category; 65A7 and 82G10 were in the same category; 12C10 and 32D6 were in one category; and 4A8 and 16C2 were in one category (**Supplementary Figure 1A**). The results of the homology analysis of the heavy and light chain amino acid sequences of the antibodies showed that the heavy and light chain sequence homology was relatively low, especially that of the heavy chain, which indicated a high diversity of anti-CD38 antibodies (**Supplementary Figures 1B–D**).

### 3.2 High-affinity anti-CD38 antibodies that specifically target CD38

The affinity of anti-CD38 antibodies for human CD38 was assessed using BLI technology. The affinity and binding kinetics were determined to calculate the dissociation constants and determine the KD. The binding of CD38 antibodies to various hematologic tumor cell lines (Raji, Daudi, L-1236, NCI-H929, Reh, and MOLM-13) *in vitro* was assessed via flow cytometry. The CD38 expression profile of the tumor cell lines was analyzed using the anti-CD38 mouse IgG1 antibody 26B4 (**Supplementary Figure 2**). The EC50 was defined as the antibody concentration that resulted in an approximately 50% response when the antibodies were specifically bound to CD38-expressing target cells in these experiments.

The calculated KD values for all anti-CD38 antibodies (except 65A7) that bound to purified human CD38 were lower than the corresponding KD values for daratumumab and isatuximab (**Supplementary Figure 3**). The results of flow cytometry assays showed that all anti-CD38 antibodies bound to CHO-CD38 cells in a concentration-dependent manner, but they did not bind to CHO cells (**Supplementary Figures 4A, B**). However, the anti-CD38 antibodies that bound to monkey CD38 included only 65A7, 12C10, 32D6, 82G10, 4A8, and 16C2 (**Supplementary Figure 4C**). The results of the flow cytometry assays showed that all anti-CD38 antibodies bound to cells in various CD38^+^ tumor cell lines in a concentration-dependent manner; 35G5 and 25B5 exhibited a stronger binding ability (**Supplementary Figures 5A–F**).

### 3.3 Anti-CD38 antibodies exhibited potent antitumor effects through Fc-dependent effector functions in malignant cells expressing CD38

**Figure 1:**
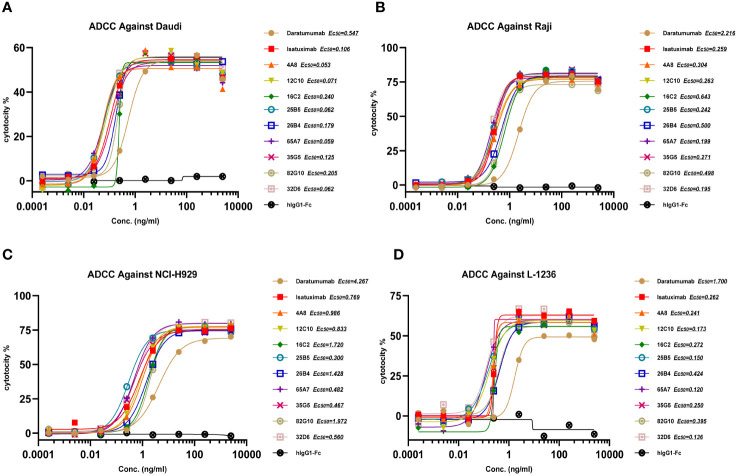
Anti-CD38 antibodies exhibited ADCC in hematologic cancer cell lines in a concentration-dependent manner. **(A, B)** Anti-CD38 antibodies exhibited ADCC activity against non-Hodgkin’s lymphoma cells (Daudi and Raji). **(C)** Anti-CD38 antibodies exhibited ADCC against multiple myeloma cells (NCI-H929). **(D)** Anti-CD38 antibodies exhibited ADCC in Hodgkin’s lymphoma cells (L-1236).

**Figure 2:**
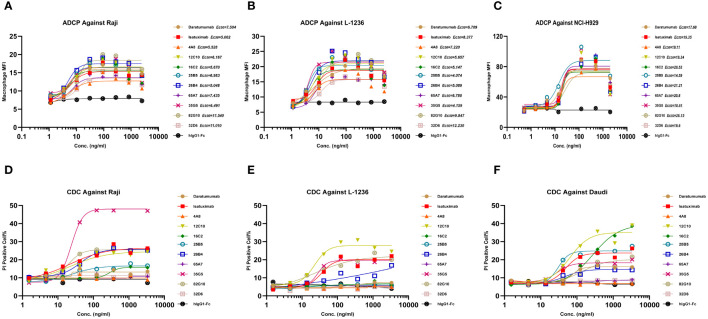
Anti-CD38 antibodies exhibited ADCP and CDC in hematologic cancer cell lines in a concentration-dependent manner. **(A–C)** Anti-CD38 antibodies were used to detect ADCP in Raji, L-1236, and NCI-H929 cells, respectively, as determined by flow cytometry. **(D–F)** Anti-CD38 antibodies were used to detect CDC against Raji, L-1236, and Daudi cells, respectively, as determined by flow cytometry.

In the development of therapeutic antibodies targeting anti-CD38, our focus was on prioritizing IgG1. This choice was driven by its high affinity for binding and activating FcγRs, leading to the potent induction of ADCP and ADCC against CD38^+^ tumor cells. IgG1 formats were selected for anti-CD38 antibodies, with the substitution of E333A, S298A, and K334A in the Fc region to enhance Fc-mediated effector functions. *In vitro*, anti-CD38 antibodies induced the lysis of CD38^+^ tumor cells through CDC, ADCP, and ADCC. Anti-CD38 antibodies exhibited strong ADCC activity against cells associated with hematological malignancies (Daudi, L-1236, Raji, and NCI-H929 cell lines) in a concentration-dependent manner *via* FcR-TANK (effector). The activity of all the anti-CD38 antibodies was similar and was significantly greater than that of daratumumab (**Figures 1A–D**). By conducting flow cytometry assays, phagocytosis was evaluated in the monocytic cell line THP-1 (effector) against the target cell lines Raji, L-1236, and NCI-H929 labeled with CFSE by measuring the green fluorescence of THP-1 cells following 2 h of incubation with anti-CD38 antibodies. The anti-CD38 antibodies also exhibited strong concentration-dependent ADCP activity against the Raji, L-1236, and NCI-H929 cell lines (**Figures 2A–C**). Antibody CDC induction was related to the density of the target protein and the inhibitory complement regulatory proteins (CD46, CD55, and CD59). Cell lysis was used as a marker for CDC, as determined by the uptake of propidium iodide (PI) via flow cytometry analysis. Hematologic cancer cells (Daudi, L-1236, Raji, and NCI-H929 cell lines) were incubated with anti-CD38 antibodies in the presence of complement-containing human serum. The CDC activity of different antibodies was different, and the CDC activity of the same antibody in different cell lines was also different. None of the anti-CD38 antibodies showed CDC activity against NCI-H929 cells (data not presented). Additionally, the 12C10, 35G5, 82G10, and 26B4 antibodies showed strong CDC activity against Raji, L-1236, and L-1236 cells (**Figures 2D–F**).

### 3.4 Anti-CD38 antibodies directly induced apoptosis of CD38-expressing lymphoma cells

**Figure 3:**
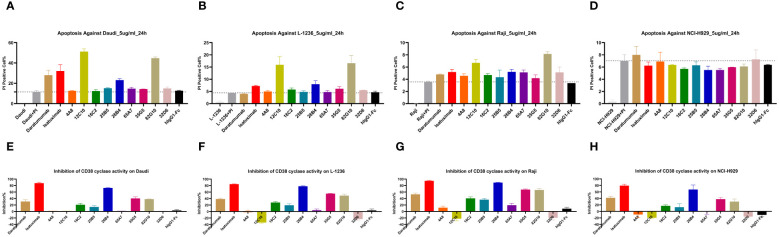
Anti-CD38 antibodies directly induced apoptosis and inhibited cellular CD38 enzymatic activity. **(A–D)** Anti-CD38 antibodies induced apoptosis in Daudi, L-1236, Raji, and NCI-H929 cells, respectively, as determined by flow cytometry. None of the antibodies showed apoptotic activity against NCI-H929 cells, whereas the 12C10 and 82G10 antibodies showed the strongest apoptotic activity against Daudi, L-1236, and Raji cells via direct induction. **(E–H)** Anti-CD38 antibodies induced CD38 enzymatic inhibition in Daudi, L-1236, Raji, and NCI-H929 cells, respectively, as determined by using NGD as a substrate. The 26B4, 35G5, and 82G10 antibodies showed the strongest ability to inhibit enzymatic cyclase activity.

Daudi, L-1236, Raji, and NCI-H929 cells were cultured with anti-CD38 antibodies for 24 h, and apoptosis was measured by PI staining. The apoptotic activity of different antibodies differed, and the activity of the same antibody in different cell lines also differed. None of the antibodies showed apoptotic activity against NCI-H929 cells. The 12C10 and 82G10 antibodies showed the strongest apoptosis-inducing activity against Daudi, L-1236, and L-1236 cells (**Figures 3A–D**).

### 3.5 Anti-CD38 antibodies inhibited cellular CD38 enzymatic activity

The effect of anti-CD38 antibodies on the ADP-ribosyl cyclase activity of CD38 was examined by incubation with CD38-expressing cells in assays using nicotinamide guanine dinucleotide (NGD) as a substrate. In assays where NGD was used as the substrate, 26B4, 35G5, and 82G10 exhibited the strongest ability to inhibit the enzymatic cyclase activity of CD38-expressing Daudi, Raji, NCJ-H929, and L-1236 cells. In contrast, the 4A8, 12C10, 65A7, and 32D6 antibodies did not influence the enzymatic cyclase activity (**Figures 3E–H**).

### 3.6 Binding of anti-CD38 antibodies to human RBCs and platelets

Peripheral blood was collected from humans and incubated with anti-CD38 antibodies *in vitro*. RBCs were identified based on their light-scattering properties using a flow cytometer. Unlike the CD47 antibody Hu5F9, even at a concentration of 100 nM, the anti-CD38 antibodies did not bind to RBCs and did not induce RBC agglutination (**Supplementary Figures 6A, B**). Human blood samples incubated with anti-CD38 antibodies were counterstained with anti-CD61 antibodies to identify platelets in whole blood. Similarly, anti-CD38 antibodies did not bind to platelets at a concentration of 100 nM (**Supplementary Figure 6C**).

### 3.7 The design, quality evaluation, and binding activity of CD38/CD47 BsAbs

**Figure 4:**
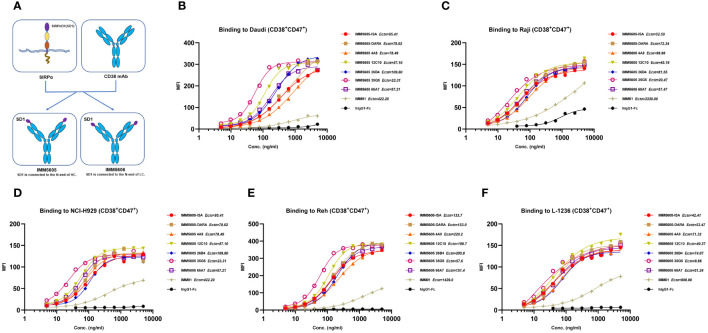
Illustration of the design of CD38/CD47 BsAbs and their binding ability. **(A)** A co-targeting CD47 and CD38 bispecific antibody (BsAb) was designed using a 2 + 2 “mAb-trap” platform. IMM5605 consists of a CD38 antibody, and two SIRPαD1 ligand trap domains connected to the N-terminus of the antibody heavy chain, while IMM5606 has SIRPαD1s connected to the N-terminus of its CD38 antibody light chains. **(B–F)** CD38/CD47 BsAbs bound to CD38+/CD47+ hematologic cancer cells (Daudi, Raji, NCI-H929, Reh, and L-1236) in a concentration-dependent manner, and the binding ability of BsAbs was significantly greater than that of IMM01.

**Figure 5:**
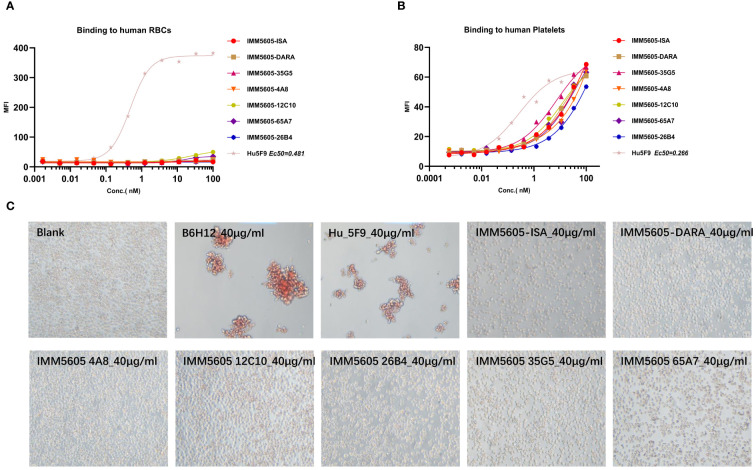
CD38/CD47 BsAb-induced RBC agglutination and binding analysis. **(A, B)** CD38/CD47 BsAbs bound to RBCs and platelets, respectively, as determined by flow cytometry assays. **(C)** CD38/CD47 BsAbs induced RBC agglutination. Unlike the CD47 antibody Hu5F9, even at a concentration of 100 nM, CD38/CD47 BsAbs did not bind to RBCs, but all BsAbs could weakly bind to platelets, and all BsAbs did not induce RBC agglutination at a concentration of 40 μg/ml.

Based on *in vitro* activity data, we selected five anti-CD38 antibodies (4A8, 12C10, 26B4, 35G5, and 65A7) for the development of CD38/CD47 BsAbs using a 2 + 2 “mAb-trap” platform (**Table 1**). The CD38/CD47 BsAbs consisted of the anti-CD38 antibody and the SIRPα IgV domain (SIRPα domain 1, SD1), connected to the N-terminus of the heavy chain (IMM5605) or the light chain (IMM5606) of the antibody (**Figure 4A**). We also used daratumumab and isatuximab antibody frameworks to design CD38/CD47 BsAbs as controls. IMM5605-ISA and IMM5605-DARA were connected via SD1 to the N-terminus of the heavy chain of isatuximab and daratumumab, respectively. The purity of the CD38/CD47 BsAbs was evaluated by HPLC-SEC and SDS–PAGE, and the results showed that the purity was greater than 90% (**Supplementary Figures 7A, B**). We also used BLI technology to evaluate the affinity of CD38/CD47 BsAbs for CD47 and CD38 targets. The findings revealed that the affinity of the BsAbs for the CD38 target was significantly greater (except for 65A7) than that of the controls IMM5605-ISA and IMM5605-DARA. The affinity of the CD38/CD47 BsAbs for the CD38 target was significantly greater than that for the CD47 target, except for 65A7 (**Supplementary Figures 8**, **9**). These results indicated that CD38/CD47 BsAbs preferentially bound to CD38^+^/CD47^+^ tumors and avoided binding to CD47^+^ normal tissues, thus reducing the potential for off-target effects. The results of the flow cytometry analysis showed that compared to IMM01 (SIRPα Fc fusion protein), all CD38/CD47 BsAbs had a greater ability to bind to CD38^+^ tumor cell lines in a concentration-dependent manner, and the binding ability of 35G5 and 12C10 was greater than that of the other BsAbs (**Figures 4B–F**). We also analyzed the binding ability of CD38/CD47 BsAbs to human RBCs and found that even at a concentration of 100 nM, BsAbs had very little binding to RBCs, but Hu5F9 strongly bound to RBCs (**Figure 5A**). All CD38/CD47 BsAbs could bind to platelets in a dose-dependent manner but were weaker than Hu5F9 (**Figure 5B**). More importantly, none of the CD38/CD47 BsAbs induced RBC agglutination (**Figure 5C**).

**Table 1:** Summary of the data on anti-CD38 antibodies.

Clones ID	Epitope	Cross with cyno CD38	Affinity (BLI)	Inhibition%				Binding (Ec50, ng/ml)							Apoptosis (%)				CDC (Ec50, ng/ml)				ADCC (Ec50, ng/ml)				ADCP (Ec50, ng/ml)		
				Daudi	Raji	NCI-H929	L-1236	CHO-CD38	CHO	Raji	Daudi	NCI-H929	L-1236	Reh	Daudi	L-1236	Raji	NCI-H929	Daudi	Raji	L-1236	NCI-H929	Daudi	Raji	L-1236	NCI-H929	Raji	L-1236	NCI-H929
Daratumumab	/	NB	4.47E-08	31	53	41.8	38.6	327.4	NB	55.4	54.8	125.7	42.9	69.8	16.5	-0.4	4.8	no	75.7	no	no	no	0.547	2.216	1.7	4.267	7.5	6.71	17.68
Isatuximab	/	NB	1.09E-08	87.6	94.6	79.2	84.5	312.7	NB	49.2	45.6	82.3	38	63	20.5	2.9	5.2	no	48.5	21.7	34.7	no	0.106	0.259	0.262	0.769	5.06	8.38	19.35
**4A8**	**D**	**Binding**	**< 1.00E-012**	**1.5**	**11.8**	**-9.5**	**0.3**	**866.7**	**NB**	**203.1**	**161.7**	**485**	**298.7**	**590.5**	**1**	**0.6**	**4.6**	**no**	**no**	**no**	**no**	**no**	**0.053**	**0.304**	**0.241**	**0.986**	**5.53**	**7.22**	**19.11**
**12C10**	**C**	**Binding**	**< 1.00E-012**	**1.5**	**-22.3**	**-19.6**	**-33.8**	**264.4**	**NB**	**41.4**	**46.8**	**58.3**	**33.8**	**94.9**	**39.7**	**11.6**	**6.7**	**no**	**63.9**	**22.1**	**22.4**	**no**	**0.071**	**0.263**	**0.173**	**0.833**	**6.17**	**5.66**	**19.34**
16C2	D	Binding	< 1.00E-012	20.7	40.4	17	28.1	483.3	NB	76.5	108	160.6	NA	527.8	0.8	1.4	4.7	no	277.1	283.8	no	no	0.24	0.643	0.272	1.72	5.67	5.15	20.53
25B5	A	NB	9.53E-09	14	36.5	13	19.4	135	NB	20.6	12.8	19	NA	NA	3.6	0.5	4.3	no	30.4	26	no	no	0.062	0.242	0.15	0.3	6.98	4.07	14.59
**26B4**	**A**	**NB**	**< 1.00E-012**	**72.7**	**89.3**	**67.5**	**78**	**245**	**NB**	**41.1**	**47.6**	**68**	**33.3**	**63.8**	**11.6**	**3.6**	**5.2**	**no**	**46.6**	**42.7**	**NA**	**no**	**0.179**	**0.5**	**0.424**	**1.428**	**5.05**	**5.09**	**21.21**
**65A7**	**B**	**Binding**	**1.90E-08**	**1.5**	**19.5**	**-0.9**	**4.4**	**242.1**	**NB**	**32.1**	**42.2**	**46.7**	**35**	**62**	**3.2**	**0.4**	**5.1**	**no**	**no**	**no**	**no**	**no**	**0.059**	**0.199**	**0.12**	**0.482**	**7.44**	**6.78**	**20.8**
**35G5**	**A**	**NB**	**< 1.00E-012**	**40.4**	**67.7**	**37.7**	**55.2**	**188.5**	**NB**	**22.9**	**31.1**	**25.9**	**17.3**	**34.2**	**2.8**	**1.8**	**4.2**	**no**	**28.9**	**23.7**	**27.8**	**no**	**0.125**	**0.271**	**0.25**	**0.467**	**6.49**	**4.16**	**18.61**
82G10	B	Binding	< 1.00E-012	38	66.6	30.3	49.2	667.9	NB	117	91.7	252.6	145.4	173	33.2	12.2	8.2	no	139.7	20.6	23.2	no	0.205	0.498	0.395	1.972	11.54	9.85	26.15
32D6	C	Binding	8.13E-09	1.5	-19.4	-15.7	-23.2	194.9	NB	42.8	14.7	60.2	39.1	NA	3.3	1.2	5.1	no	no	no	no	no	0.062	0.195	0.136	0.56	11.01	12.23	19.6

Based on *in vitro* activity data, five anti-CD38 antibodies (4A8, 12C10, 26B4, 35G5, and 65A7) were selected for the development of CD38/CD47 BsAbs via a 2 + 2 “mAb-trap” platform.

The bold fonts represent the clones that were selected for the development of CD38/CD47 BsAbs.

### 3.8 CD38/CD47 BsAbs simultaneously bound to CD47 and CD38 targets

**Figure 6:**
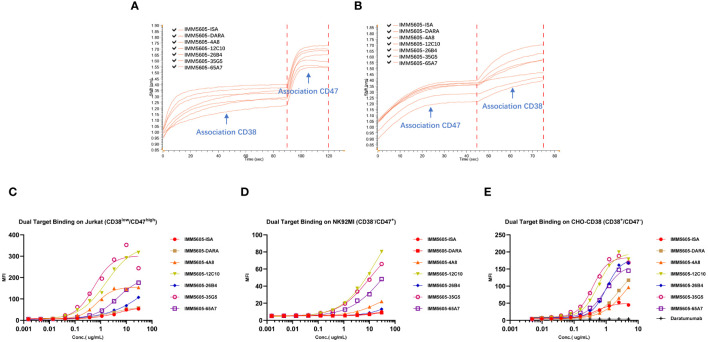
The dual-target binding of CD38/CD47 BsAbs was analyzed by BLI and flow cytometry. **(A)** The CD38/CD47 BsAbs first bound to the CD38 target and then to the CD47 target. **(B)** The CD38/CD47 BsAbs first bound to the CD47 target and then to the CD38 target. All CD38/CD47 BsAbs can simultaneously bind to the CD47 and CD38 targets, as determined by BLI assays. **(C)** The CD38/CD47 BsAbs first bound to the CD38low/CD47+ Jurkat cells and then to the CD38 protein with an mFc tag. **(D)** The CD38/CD47 BsAbs first bound to the CD38-/CD47+ NK92MI cells and then to the CD38 protein with an mFc tag. **(E)** The CD38/CD47 BsAbs first bound to CD38+/CD47- CHO-CD38 and then to the CD47 protein with an mFc tag. All CD38/CD47 BsAbs can simultaneously bind to the CD47 and CD38 targets, as determined by flow cytometry assays. 35G5 and 12C10 exhibited the strongest ability to simultaneously bind to both CD47 and CD38.

We used BLI technology to evaluate the ability of CD38/CD47 BsAbs to simultaneously bind purified CD47 and CD38. The results indicated that, irrespective of whether BsAbs first bound to CD47 or CD38, they could bind to the other antigen (**Figures 6A, B**). We also performed a flow cytometry assay to evaluate the ability of CD38/CD47 BsAbs to simultaneously bind CD47^+^ cells and CD38^+^ cells. The results showed that BsAbs could bind to cellular CD47 and CD38 simultaneously. 35G5 and 12C10 exhibited the strongest ability to simultaneously bind to the CD47 and CD38 targets (**Figures 6C–E**).

### 3.9 The CD38/CD47 BsAbs preferentially bound to CD38^+^/CD47^+^ cells

**Figure 7:**
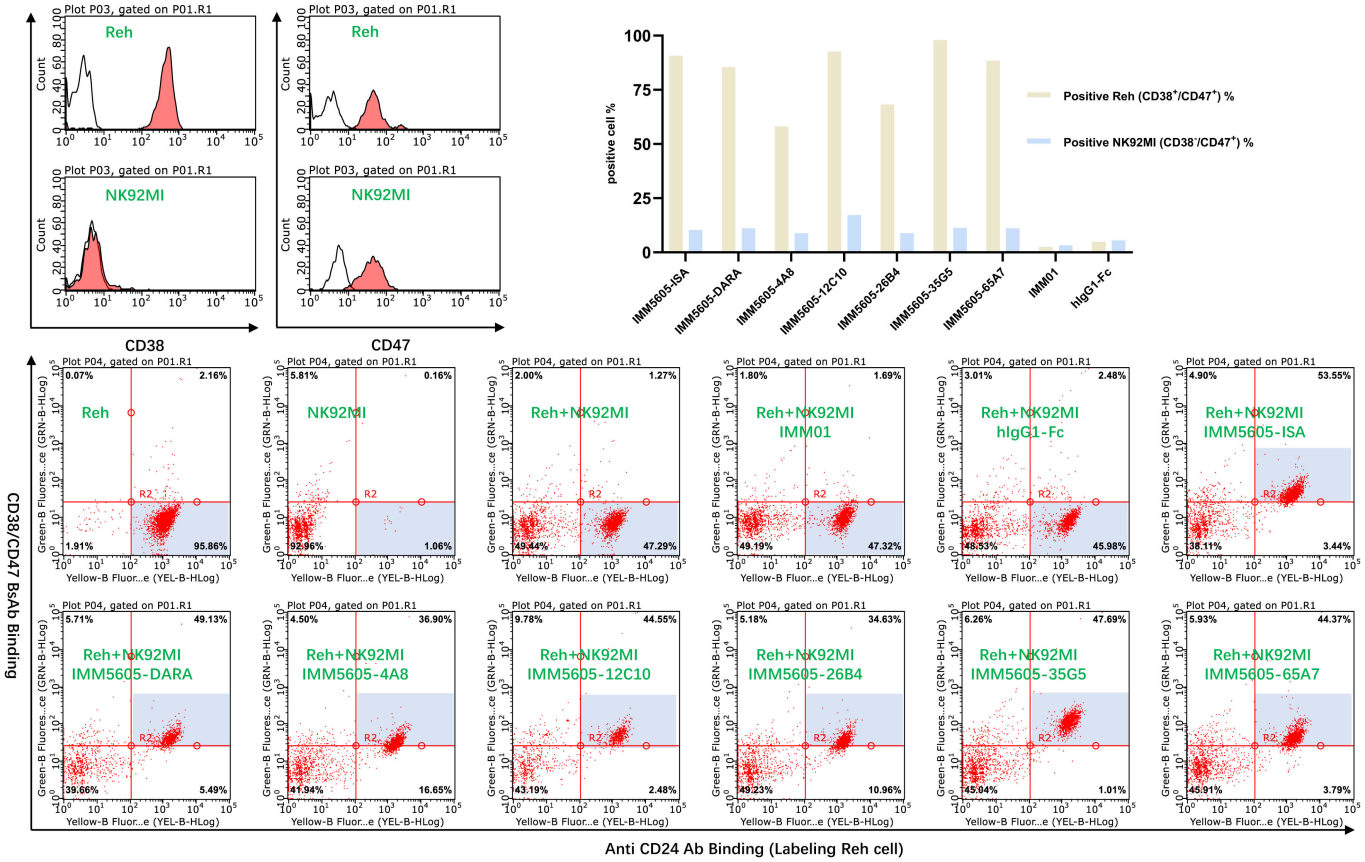
The off-target effects of the CD38/CD47 BsAbs were analyzed by flow cytometry. CD38/CD47 BsAbs preferentially bound to CD38^+^/CD47^+^ cells, as the CD38/CD47 BsAbs consisted of lower-affinity CD47 and higher-affinity CD38 binding arms.

As CD47 is a ubiquitously expressed molecule, we designed a panel of CD38/CD47 BsAbs, which consisted of a CD47-binding arm with lower affinity than the CD38-binding arm. To assess the specificity of the CD38/CD47 BsAbs, we obtained the binding profile of CD38^+^/CD47^+^ Reh cells mixed with CD38^-^/CD47^+^ NK92MI cells at a 1:1 ratio by performing flow cytometry assays. CD38/CD47 BsAbs preferentially bound to CD38^+^/CD47^+^ Reh cells, indicating that compared to CD47 therapeutic Abs, BsAbs have lower potential for on-target, off-tumor effects *in vivo* (**Figure 7**).

### 3.10 The CD38/CD47 BsAbs blocked the interaction between CD47 and SIRPα and inhibited the enzymatic activity of CD38

**Figure 8:**
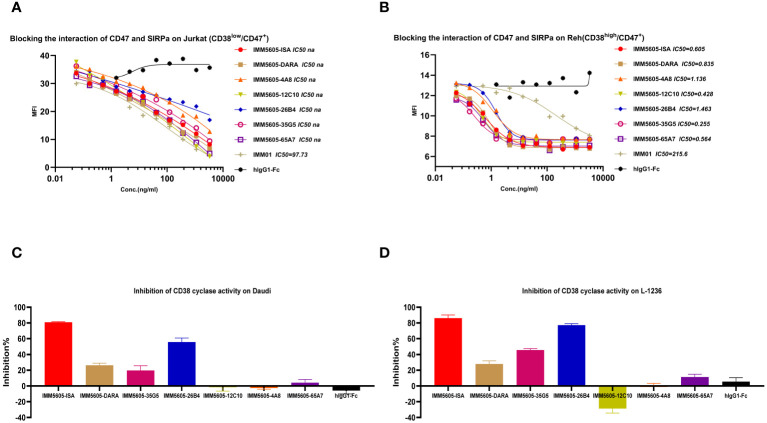
CD38/CD47 BsAbs blocked CD47/SIRPα signaling and inhibited cellular CD38 enzymatic activity. **(A, B)** CD38/CD47 BsAbs blocked the interaction of CD47/SIRPα in Jurkat cells (CD38^Low^/CD47^+^) and Reh cells (CD38^high^/CD47^+^), respectively, as determined by flow cytometry. The results revealed that the blocking activity of the CD38/CD47 BsAbs was significantly greater in the CD47 and CD38 double-positive cells. **(C, D)** CD38/CD47 BsAbs induced apoptosis in Daudi and L-1236 cells, respectively, as determined by using NGD as a substrate. The 26B4 antibody exhibited the strongest ability to inhibit enzymatic cyclase activity, followed by the 35G5 antibody.

The ability of CD38/CD47 BsAbs to block the interaction between CD47 and SIRPα was tested in a CD47^+^/CD38^low^ Jurkat cell line and a CD47^+^/CD38^+^ Reh cell line. The results indicated that the ability of BsAbs to block the interaction between CD47 and SIRPα was lower than that of the IMM01 in the Jurkat cell line (**Figure 8A**). In contrast, the blocking activity of the CD38/CD47 BsAbs was 200–500-fold greater than that of IMM01 in the Reh cell line (**Figure 8B**). Our findings showed that the CD38/CD47 BsAbs strongly blocked the CD47/SIRPα interaction in CD38/CD47 double-positive tumor cells, indicating that the inhibition of CD47/SIRPα depended on the engagement of CD38. We evaluated the inhibitory effect of CD38/CD47 BsAbs on the ADP ribosyl-cyclase enzymatic activity of cellular CD38 *in vitro* and found that 26B4 had the strongest inhibitory effect on cellular CD38 enzymatic function, followed by 35G5; however, the other BsAbs had no inhibitory effect (**Figures 8C, D**).

### 3.11 The CD38/CD47 BsAbs exhibited strong antitumor effects *in vitro*

**Figure 9:**
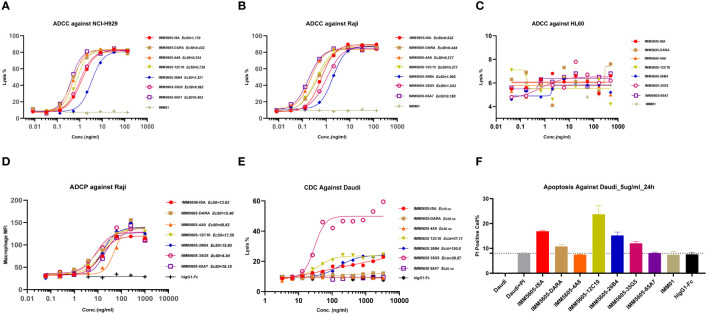
CD38/CD47 BsAbs exhibited ADCC, ADCP, CDC and apoptosis in hematologic cancer cell lines, as determined by flow cytometry. **(A–C)** CD38/CD47 BsAbs exhibited ADCC in NCI-H929, Raji, and HL-60 cells, respectively. The data revealed that the CD38/CD47 BsAbs had no ADCC effect on HL-60 cells (CD38-/CD47+), which indicated that the CD38 arm was the major contributor to ADCC. **(D)** CD38/CD47 BsAbs exhibited ADCP in Raji cells. **(E)** CD38/CD47 BsAbs exhibited CDC in Daudi cells. The results showed that 12C10, 35G5, and 26B4 exhibited relatively weak CDC effects. **(F)** The ability of CD38/CD47 BsAbs to directly induce apoptosis in Daudi cells was assessed via flow cytometry, which indicated that 12C10 exhibited the strongest ability to induce apoptosis.

We also selected IgG1 for designing CD38/CD47 BsAbs, as well as an engineered Fc domain (S298A, E333A, and K334A) for enhancing Fc-dependent effector functions. We used an engineered NK cell line and primary PBMC as effector cells to evaluate ADCC activity of CD38/CD47 BsAbs. The CD38/CD47 BsAbs exhibited potent ADCC activity against CD47^+^/CD38^+^ Raji and NCI-H929 cells in a concentration-dependent manner. Except for 26B4, which had a lower activity than the other BsAbs, the difference in activity between the other BsAbs was 1–3 times (**Figures 9A, B**; **Supplementary Figure 10**). Additionally, the BsAbs did not exhibit an ADCC effect on HL-60 (CD47^+^/CD38^–^) cells, which indicated that the CD38 arm was the major contributor to ADCC (**Figure 9C**). No major ADCC was detected in normal cells expressing CD47, suggesting that the BsAbs exhibited less on-target, off-tumor toxicity. The evaluation of phagocytosis was carried out in the monocytic cell line THP-1 (effector) against the target cell lines Raji and NCI-H929 labeled with CFSE by measuring the THP-1 green fluorescence after 2 hours of incubation with CD38/CD47 BsAbs. The BsAbs also exhibited strong ADCP activity against the Raji and NCI-H929 cell lines in a concentration-dependent manner and showed ADCP activity similar to that exhibited by the IMM5605-ISA and IMM5605-DARA controls (**Figure 9D**). We assessed the CDC effects of CD38/CD47 BsAbs against Daudi cells and found that 12C10, 35G5, and 26B4 exhibited relatively weak CDC effects (**Figure 9E**). The ability of CD38/CD47 BsAbs to directly induce apoptosis in the Daudi cell line was evaluated by flow cytometry assays, and the results showed that 12C10 exhibited the strongest ability to induce apoptosis (**Figure 9F**).

### 3.12 CD38/CD47 BsAbs reduced the tumor burden in a multiple myeloma xenograft mouse model

**Figure 10:**
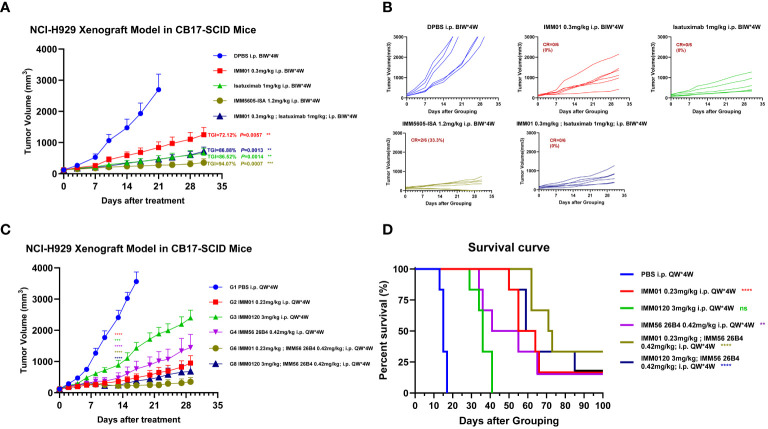
Evaluation of the *in vivo* efficacy of the anti-CD38 antibody combined with the CD47 blocker in the NCI-H929 xenograft mouse model. **(A, B)** *In vivo* efficacy of IMM01, isatuximab, combination treatment, and IMM5605-ISA (based on isatuximab) in the NCI-H929 xenograft mouse model. The results showed that IMM5605-ISA was significantly more efficacious at controlling tumor growth than other therapeutics. **(C, D)** *In vivo* efficacy of IMM01 or IMM0120 combined with the anti-CD38 antibody 26B4 in the NCI-H929 xenograft model. All treatment groups demonstrated therapeutic effects to different degrees. In the NCI-H929 tumor cell xenograft model, targeting both CD47 and CD38 significantly prolonged survival, whereas targeting only one of them was insufficient. The p values were calculated based on different groups of tumor volume using the vehicle group as the control (Student’s t-test). The p values were calculated based on the survival times of the different groups using the log-rank (Mantel–Cox) test, and the vehicle group was used as the control. The p values are as follows: *, *P* < 0.05; **, *P* < 0.01; ***, *P* < 0.001; ****, *P* < 0.0001; n.s., not significant.

**Figure 11:**
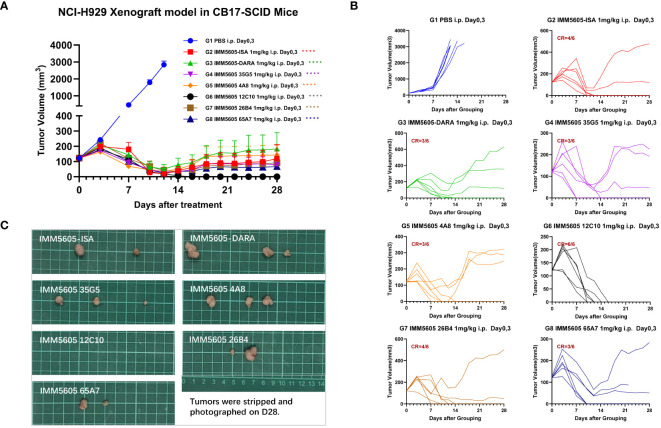
Assessment of the *in vivo* efficacy of CD38/CD47 BsAbs in the NCI-H929 xenograft mouse model. **(A)** Tumor growth curves of the subcutaneous NCI-H929 xenograft model in female CB17-SCID mice in all groups after administration. **(B)** Tumor growth curves of individual mice in all groups after administration. **(C)** Tumor images at the endpoint of the experiment. All CD38/CD47 BsAbs (1 mg/kg, D0/D3, IP) demonstrated high antitumor efficacy, among which IMM5605–12C10 exhibited the highest complete response rate and eliminated all tumors in all mice. ****p < 0.0001.

We next assessed the *in vivo* antitumor killing efficacy of BsAbs targeting CD47 and CD38 in a mouse xenograft model using CB17-SCID mice implanted with the NCI-H929 multiple myeloma cell line. IMM5605-ISA was significantly more efficacious in controlling tumor growth, with a D21 tumor growth inhibition (TGI) of 94.07%, than was the control. IMM01, isatuximab, and their combination had partial effects on tumor growth, with final TGIs of 72.12%, 86.52%, and 86.88%, respectively (**Figures 10A, B**). Next, we evaluated the therapeutic effects of IMM01 (SIRPαD1 Fc fusion protein, IgG1) or IMM0120 (SIRPαD1 Fc fusion protein, IgG4) combined with the anti-CD38 antibody 26B4 in an NCI-H929 xenograft model. Tumor growth was monitored beyond the end of the treatment period, and the results indicated that the combination treatment decreased tumor growth for a longer period than any single-drug treatment (**Figures 10C, D**). These results confirmed that co-targeting CD47 and CD38 greatly enhanced antitumor efficacy. Based on these findings, we designed BsAbs targeting CD47 and CD38 using the 2 + 2 “mAb trap” platform and evaluated the effectiveness of the BsAbs through *in vitro* and *in vivo* experiments. The NCI-H929 tumor xenograft model was used to evaluate the efficacy of the CD38/CD47 BsAbs, including IMM5605-ISA, IMM5605-DARA, IMM5605–4A8, 12C10, 26B4, 35G5, and 65A7. All CD38/CD47 BsAbs (1 mg/kg, D0/D3, IP) showed high antitumor efficacy, among which IMM5605–12C10 exhibited the highest complete response rate and eradicated all tumors (**Figures 11A–C**).

## 4 Discussion

The number of patients with hematological malignancies has increased in the last 30 years, and hematological malignancies account for approximately 9% of all cases related to cancer. Currently, chemotherapy and immunotherapy are still the two main treatment methods for hematological malignancies (24). The treatment of hematological malignancies traditionally relies on chemotherapy regimens. However, although chemotherapy can benefit some patients to some extent, patients still cannot achieve complete remission, and new problems, such as relapse and drug resistance, have emerged. The clinical application of antibodies has revolutionized the treatment of CD38-positive hematologic malignancies, especially relapsed/refractory disease, for which chemotherapeutic treatment is unsatisfactory (25). In multiple myeloma (MM), CD38 is an important target for monoclonal antibody therapy because MM plasma cells have increased CD38 expression compared with that in normal cells. However, CD38 is also expressed in diseases other than MM, such as chronic lymphocytic leukemia, acute leukemia, and various lymphomas (follicular cells, mantle cells, and diffuse large B-cell lymphomas) (26–28). MM is incurable, although several classes of drugs, such as proteasome inhibitors, immunomodulatory agents, and monoclonal antibodies, are available. After non-Hodgkin’s lymphoma, MM is the 2^nd^ most common hematologic cancer, with >100,000 new cases annually (29). Daratumumab is a human IgG1 monoclonal antibody from Janssen that binds to CD38 (first-in-class) and is utilized for treating MM, lymphoma, leukemia, and systemic AL amyloidosis. Daratumumab can have various effects, such as ADCC, direct cytotoxicity, CDC, and ADCP. It stimulates apoptosis induced by the Fc gamma receptor (30, 31). Daratumumab can eliminate highly immunosuppressive subsets of Tregs, Bregs, and MDSCs (6). Isatuximab was developed by Sanofi. It is a chimeric humanized IgG1 monoclonal antibody that binds a specific epitope on CD38, a human cell surface antigen. It has been authorized for treating RRMM and has Fc-dependent and Fc-independent underlying mechanisms. Fc-dependent mechanisms include ADCP, ADCC, and CDC (32), whereas Fc-independent processes involve direct *in vitro* cytotoxicity to MM cells via lysosome-induced nonapoptotic death and caspase-dependent apoptosis (33). Furthermore, isatuximab has an *in vitro* immunomodulatory impact, indirectly controlling MM tumor growth. Some of its effects include MM cell lysis by direct NK or CD8^+^ cell activation and CD38^+^ Treg cell suppression. Isatuximab can reduce the immunosuppressive microenvironment surrounding tumors by suppressing NK and CD8+ T-cell inhibition (32, 34, 35). In this study, we obtained 9 ideal anti-CD38 antibodies by standard hybridoma technology and compared them with control antibodies (daratumumab and isatuximab); however, the variable region sequences and epitopes of these antibodies were not completely consistent. We evaluated the ability of anti-CD38 antibodies to kill CD38^+^ hematologic cancer cell lines through ADCC, CDC, ADCP, and apoptosis induction using *in vitro* assays and inhibited CD38 enzymatic activity (**Table 1**). The 12C10 antibody showed the strongest apoptosis-inducing activity, while 26B4 exhibited the strongest ability to inhibit enzymatic cyclase activity. Unlike the anti-CD47 antibody Hu5F9, the anti-CD38 antibodies did not bind to RBCs and did not induce RBC agglutination.

Macrophages express SIRPα, which binds CD47, a ubiquitously expressed protein that induces a “do not eat me” signal. This interaction is hijacked by cancer cells through the upregulation of CD47 expression on their surface, which balances prophagocytic signals and enhances the ability of cancer cells to evade innate immunity (14). CD47 is crucially linked with hematologic malignancies, such as non-Hodgkin lymphoma (NHL), acute myeloid leukemia (AML), lymphoblastic lymphoma/acute lymphoblastic leukemia (LBL/ALL), and multiple myeloma (MM) (36, 37). Therefore, inhibiting the interaction of CD47/SIRPα can be an effective strategy for enhancing the phagocytic clearance of tumor cells from the body. Various therapeutic drugs targeting CD47, including SIRPα-related fusion proteins, BsAbs, and anti-CD47 mAbs that bind CD47 and other molecules, are being developed. Therapeutic agents targeting CD47 are difficult to develop due to the ubiquitous expression pattern of the target on healthy cells. Therefore, CD47-targeting monospecific agents have poor pharmacokinetic characteristics because of their target-mediated drug disposition and toxic effects, such as anemia.

Although the treatment of lymphomas, leukemias, and multiple myelomas has markedly advanced, treatment resistance linked with clinical and molecular relapse remains very common. The treatment of refractory and relapsed disease is very complex, and failure to control the disease at this stage causes mortality in individuals with hematologic malignancies. Novel compounds and cotreatments are required to improve disease control, prevent myeloma-associated complications, delay disease progression, and ultimately improve survival. Therapeutic agents with improved or alternative target specificity and novel combination strategies that can stimulate multiple cytotoxic processes are required to overcome resistance to available therapeutics and further improve patient outcomes.

**Figure 12:**
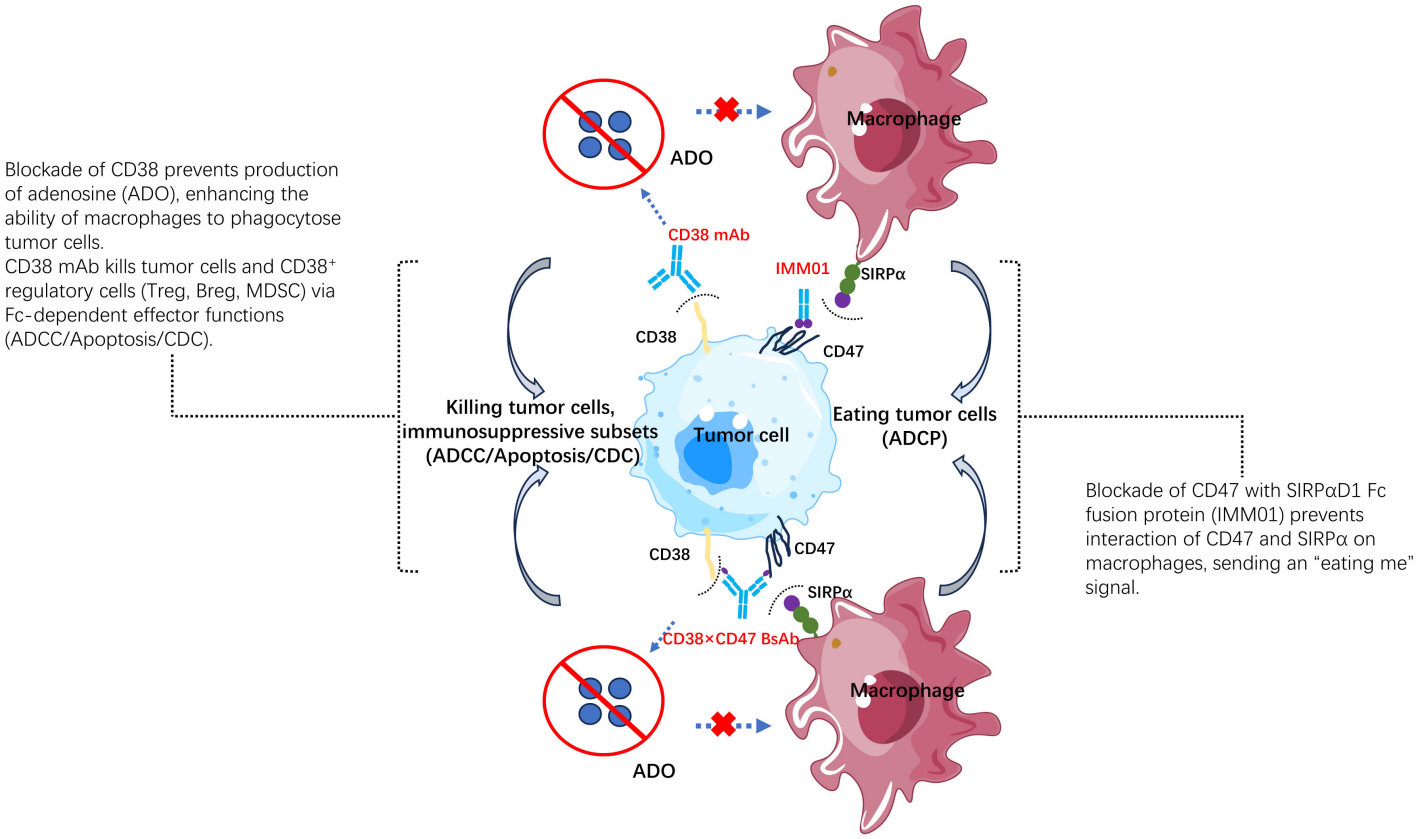
The mechanism of action of the CD38/CD47 BsAb. IMM5605 has 4 different mechanisms of action: 1) producing antibody-dependent cellular cytotoxicity (ADCC), antibody-dependent cellular phagocytosis (ADCP), complement-dependent cytotoxicity (CDC), directly inducing apoptosis via Fc gamma receptor; 2) elimination of CD38+ immunosuppressive regulatory T (Tregs) and B (Bregs) cells, as well as myeloid-derived suppressor cells (MDSCs); 3) blocking inhibitory signal from CD47/SIRPα interaction, activating macrophages, and sending an “eat tumor cell” signal. 4) blocking CD38 enzymatic activity, preventing adenosine production, and reducing inhibition in tumor microenvironment.

In this context, an enhanced CD38- and CD47-targeted therapeutic approach that can promote innate immune cell-induced tumor killing potential and overcome anti-CD38 antibody resistance mechanisms may be effective in treating hematologic malignancies. Our CD38/CD47 BsAbs (IMM5605) were 2 + 2 bispecific antibodies with CD47 and CD38 targeting domains and an engineered Fc domain to enhance ADCC, ADCP, CDC and apoptosis induction and exhibited high CD38 binding affinity, low CD47 binding affinity, negligible affinity for red blood cells (RBCs) and no induction of RBC agglutination (**Figure 12**). Using flow cytometry and biolayer interferometry (BLI), we confirmed that our CD38/CD47 BsAbs can simultaneously bind to both CD38 and CD47 and that binding to either antigen first does not prevent binding to the other antigen subsequently. Conversely, in CD38/CD47 double-positive Reh cells, our BsAbs had a 200- to 500-fold greater inhibitory effect on the CD47/SIRPα interaction than the same monospecific CD47 ligand trap molecule, indicating that the inhibition of CD47/SIRPα by our BsAbs is dependent on the engagement of CD38 on tumor cells. In Daudi cells and L-1236 cells, IMM5605–26B4 and IMM5605–35G5 inhibited CD38 enzymatic activity, which is responsible for immunosuppressive adenosine production in the tumor microenvironment. Our BsAbs presented potent dose-dependent ADCC activity against CD47^+^/CD38^+^ Raji and NCI-H929 cells but negligible ADCC activity against CD47^+^/CD38^–^ HL-60 cells, indicating low off-tumor toxicity. Similarly, our BsAbs also exhibited potent ADCP activity against Raji cells, varied degrees of CDC activity and direct induction of apoptosis in Daudi cells. In a mouse xenograft model harboring NCI-H929 multiple myeloma cells, our BsAbs inhibited tumor growth more potently than the monospecific anti-CD47 ligand trap, anti-CD38 (isatuximab) or combination of isatuximab and an anti-CD47 ligand trap at the same molecular dose per body weight. Notably, IMM5605–12C10 eradicated all tumors and achieved a 100% complete response rate in a group of six mice.

Limitations of this study include the utilization of cancer cell lines and NCI-H929 xenograft CB17-SICD mouse model for the *in vitro* and *in vivo* investigations, respectively. However, the authors selected various cancer cell lines (including Raji, Daudi, NCI-H929, L-1236, Reh and MOLM-13) due to the expression profile of CD38 and CD47 making them the suitable candidates for study (**Supplementary Figure 2**). The author selected NCI-H929 xenograft CB17-SICD mouse model validated the strong antitumor activity of CD38/CD47 BsAbs (**Figure 11**). CB17-SCID mouse exhibits severe symptoms of combined immunodeficiency, with loss of B and T lymphocyte function (38). This results in their adaptive immune response defected (including T cell-mediated immune response and B cell-mediated antibody formation). This means that they usually do not reject homologous transplants or xenografts, so CB17-SCID are commonly used as recipients of human tumor xenografts. At the same time, the strain has normal NK cells, macrophages, and granulocytes (38), and has complete innate immunity, which can attack transplanted cancer cells through antibodies mediated by antibody-dependent cellular cytotoxicity (ADCC) and antibody-dependent cellular phagocytosis (ADCP) (39–41). CD38/CD47 BsAbs (IMM5605) can inhibit tumor growth via ADCC and ADCP in xenograft CB17-SICD mouse models.

## 5 Conclusions

To summarize, we reported a novel strategy for treating hematologic malignancies that are CD38-positive by simultaneously targeting CD47 and CD38 via a first-in-class bispecific antibody. Because of their multiple mechanisms of action and unique design, CD38/CD47 BsAbs can increase antitumor activity better than anti-CD38 mAbs by overcoming acquired and primary tumor escape resistance mechanisms.

## Data availability statement

The original contributions presented in the study are included in the article/**Supplementary Material**. Further inquiries can be directed to the corresponding author.

## Ethics statement

Ethical approval was not required for the studies on humans in accordance with the local legislation and institutional requirements because only commercially available established cell lines were used. The animal studies were approved by the Institutional Animal Care and Use Committee (IACUC) of WuXi AppTec and PharmaLegacy Biology. The studies were conducted in accordance with the local legislation and institutional requirements. Written informed consent was obtained from the owners for the participation of their animals in this study.

## Author contributions

SoL: Formal analysis, Investigation, Writing – original draft, Writing – review & editing, Methodology, Project administration. DC: Formal analysis, Investigation, Writing – original draft, Writing – review & editing, Methodology, Project administration. YY: Formal analysis, Writing – original draft, Writing – review & editing, Methodology, Resources. HG: Formal analysis, Writing – original draft, Writing – review & editing, Resources. DL: Formal analysis, Writing – original draft, Writing – review & editing. NS: Formal analysis, Writing – original draft, Writing – review & editing. XB: Formal analysis, Writing – original draft, Writing – review & editing. KW: Formal analysis, Writing – original draft, Writing – review & editing. TL: Formal analysis, Writing – original draft, Writing – review & editing. GL: Formal analysis, Writing – original draft, Writing – review & editing. CY: Formal analysis, Writing – original draft, Writing – review & editing. WZ: Formal analysis, Writing – original draft, Writing – review & editing. LZ: Formal analysis, Writing – original draft, Writing – review & editing. GZ: Formal analysis, Writing – original draft, Writing – review & editing. LP: Formal analysis, Writing – original draft, Writing – review & editing. SiL: Formal analysis, Writing – original draft, Writing – review & editing. XT: Formal analysis, Writing – original draft, Writing – review & editing. RZ: Formal analysis, Writing – original draft, Writing – review & editing. WT: Conceptualization, Project administration, Writing – original draft, Writing – review & editing, Formal analysis, Funding acquisition.
