# Supplementary material for: Combining CD38 antibody with CD47 blockade is a promising strategy for treating hematologic malignancies expressing CD38
Source: Front Immunol. 2024 Jun 25;15:1398508. doi: 10.3389/fimmu.2024.1398508 (PMC11231100; doi:10.3389/fimmu.2024.1398508)
Supplement: Supplementary file 1 [file Presentation_1.pptx]

## Slide 1
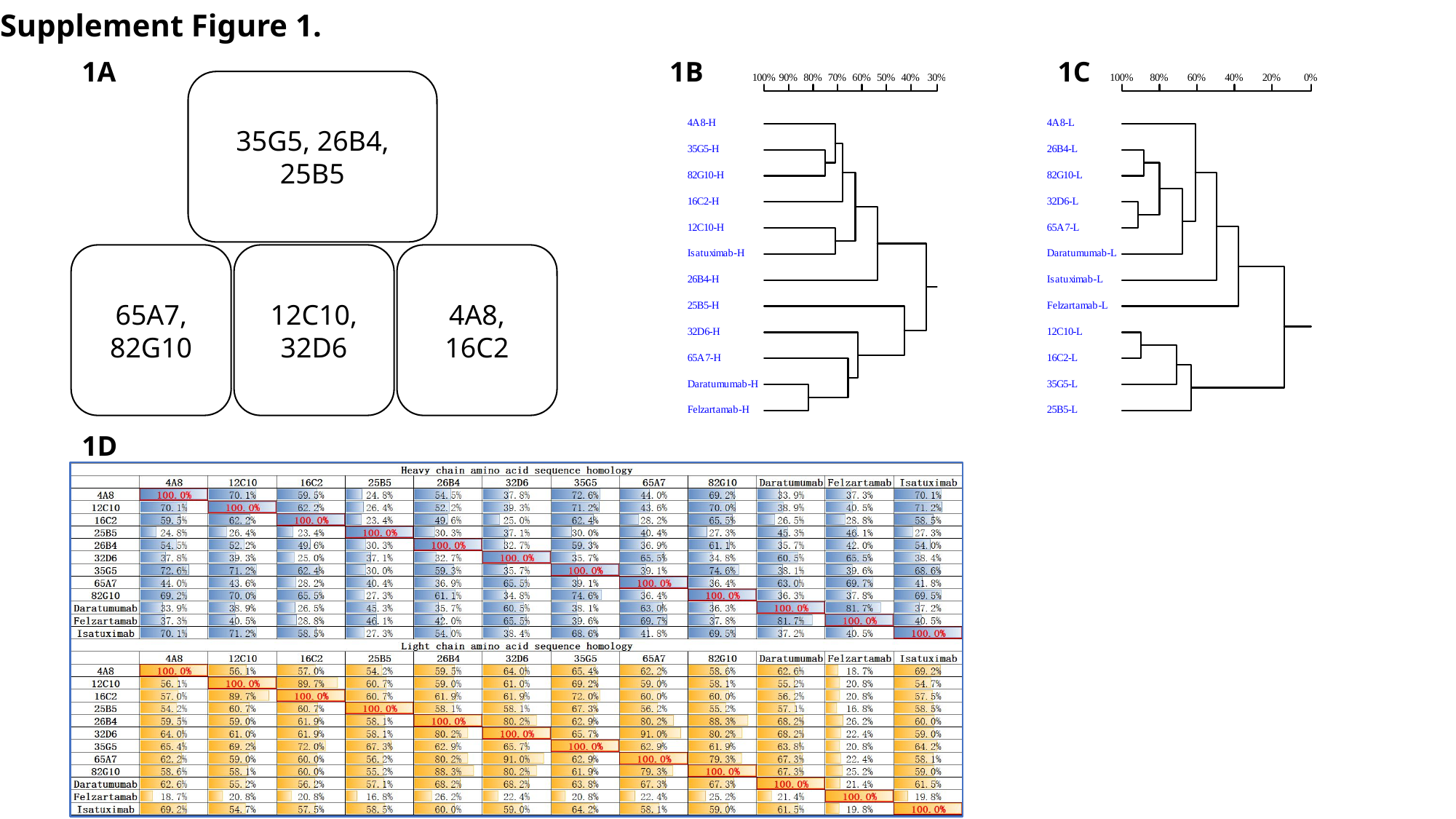

Supplement Figure 1.
1A
1B
1C
35G5, 26B4, 25B5
12C10, 32D6
4A8, 16C2
65A7, 82G10
1D

## Slide 2
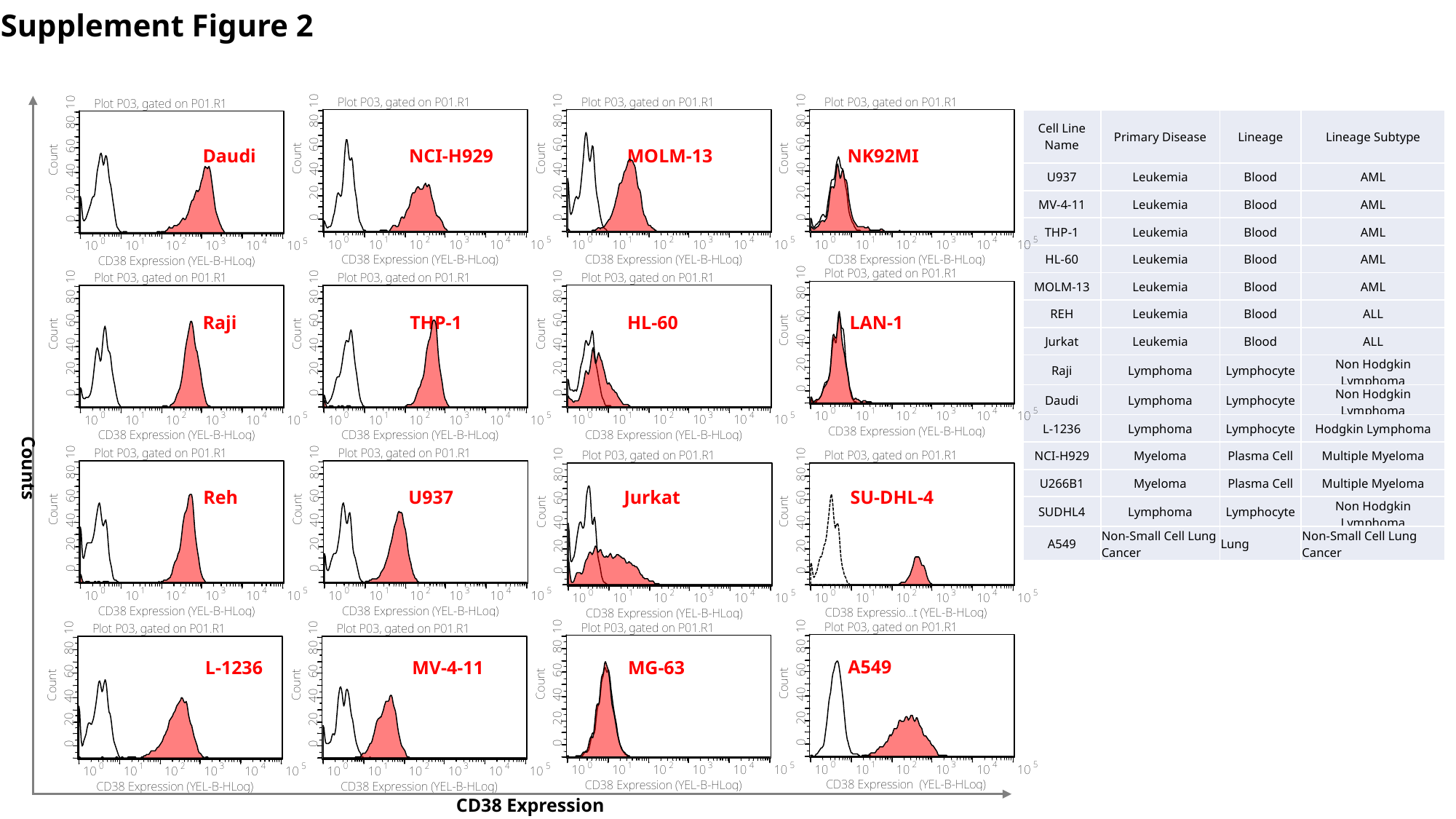

Supplement Figure 2
| Cell Line Name | Primary Disease | Lineage | Lineage Subtype |
| --- | --- | --- | --- |
| U937 | Leukemia | Blood | AML |
| MV-4-11 | Leukemia | Blood | AML |
| THP-1 | Leukemia | Blood | AML |
| HL-60 | Leukemia | Blood | AML |
| MOLM-13 | Leukemia | Blood | AML |
| REH | Leukemia | Blood | ALL |
| Jurkat | Leukemia | Blood | ALL |
| Raji | Lymphoma | Lymphocyte | Non Hodgkin Lymphoma |
| Daudi | Lymphoma | Lymphocyte | Non Hodgkin Lymphoma |
| L-1236 | Lymphoma | Lymphocyte | Hodgkin Lymphoma |
| NCI-H929 | Myeloma | Plasma Cell | Multiple Myeloma |
| U266B1 | Myeloma | Plasma Cell | Multiple Myeloma |
| SUDHL4 | Lymphoma | Lymphocyte | Non Hodgkin Lymphoma |
| A549 | Non-Small Cell Lung Cancer | Lung | Non-Small Cell Lung Cancer |
Daudi
NCI-H929
MOLM-13
NK92MI
Raji
THP-1
HL-60
LAN-1
Counts
SU-DHL-4
Reh
U937
Jurkat
A549
L-1236
MV-4-11
MG-63
CD38 Expression

## Slide 3
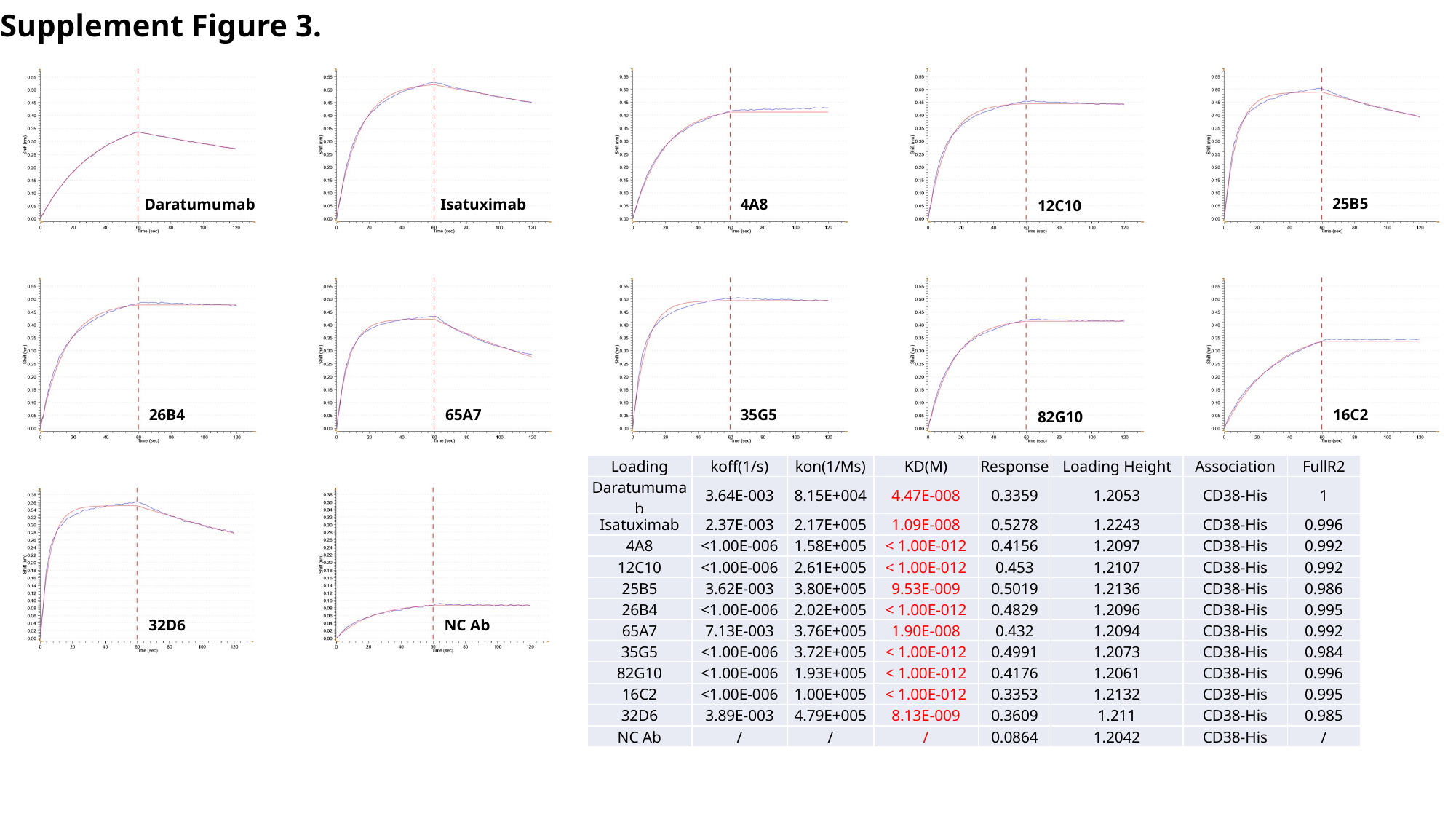

Supplement Figure 3.
25B5
4A8
Isatuximab
Daratumumab
12C10
16C2
35G5
65A7
26B4
82G10
| Loading | koff(1/s) | kon(1/Ms) | KD(M) | Response | Loading Height | Association | FullR2 |
| --- | --- | --- | --- | --- | --- | --- | --- |
| Daratumumab | 3.64E-003 | 8.15E+004 | 4.47E-008 | 0.3359 | 1.2053 | CD38-His | 1 |
| Isatuximab | 2.37E-003 | 2.17E+005 | 1.09E-008 | 0.5278 | 1.2243 | CD38-His | 0.996 |
| 4A8 | <1.00E-006 | 1.58E+005 | < 1.00E-012 | 0.4156 | 1.2097 | CD38-His | 0.992 |
| 12C10 | <1.00E-006 | 2.61E+005 | < 1.00E-012 | 0.453 | 1.2107 | CD38-His | 0.992 |
| 25B5 | 3.62E-003 | 3.80E+005 | 9.53E-009 | 0.5019 | 1.2136 | CD38-His | 0.986 |
| 26B4 | <1.00E-006 | 2.02E+005 | < 1.00E-012 | 0.4829 | 1.2096 | CD38-His | 0.995 |
| 65A7 | 7.13E-003 | 3.76E+005 | 1.90E-008 | 0.432 | 1.2094 | CD38-His | 0.992 |
| 35G5 | <1.00E-006 | 3.72E+005 | < 1.00E-012 | 0.4991 | 1.2073 | CD38-His | 0.984 |
| 82G10 | <1.00E-006 | 1.93E+005 | < 1.00E-012 | 0.4176 | 1.2061 | CD38-His | 0.996 |
| 16C2 | <1.00E-006 | 1.00E+005 | < 1.00E-012 | 0.3353 | 1.2132 | CD38-His | 0.995 |
| 32D6 | 3.89E-003 | 4.79E+005 | 8.13E-009 | 0.3609 | 1.211 | CD38-His | 0.985 |
| NC Ab | / | / | / | 0.0864 | 1.2042 | CD38-His | / |
NC Ab
32D6

## Slide 4
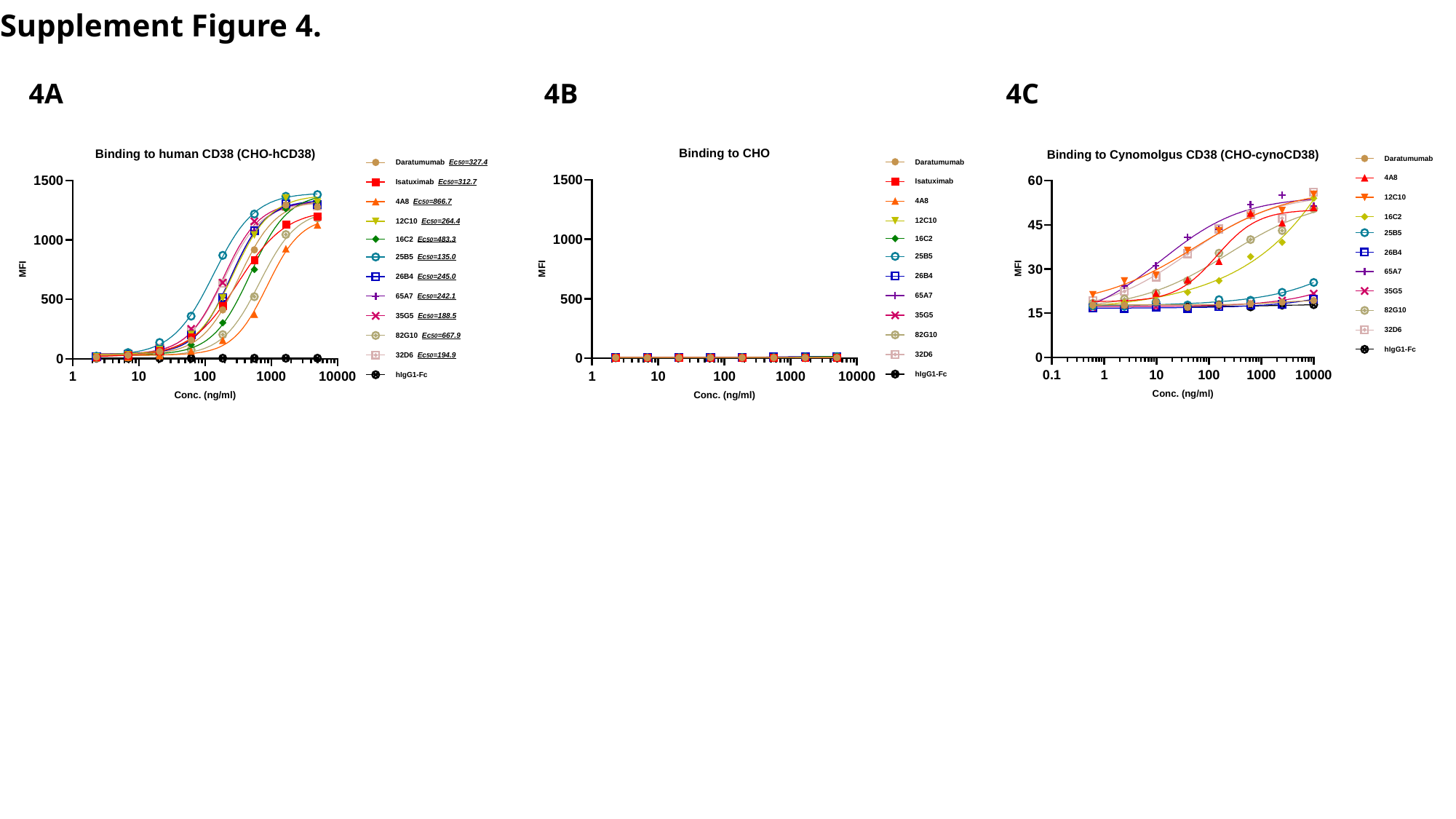

Supplement Figure 4.
4A
4B
4C

## Slide 5
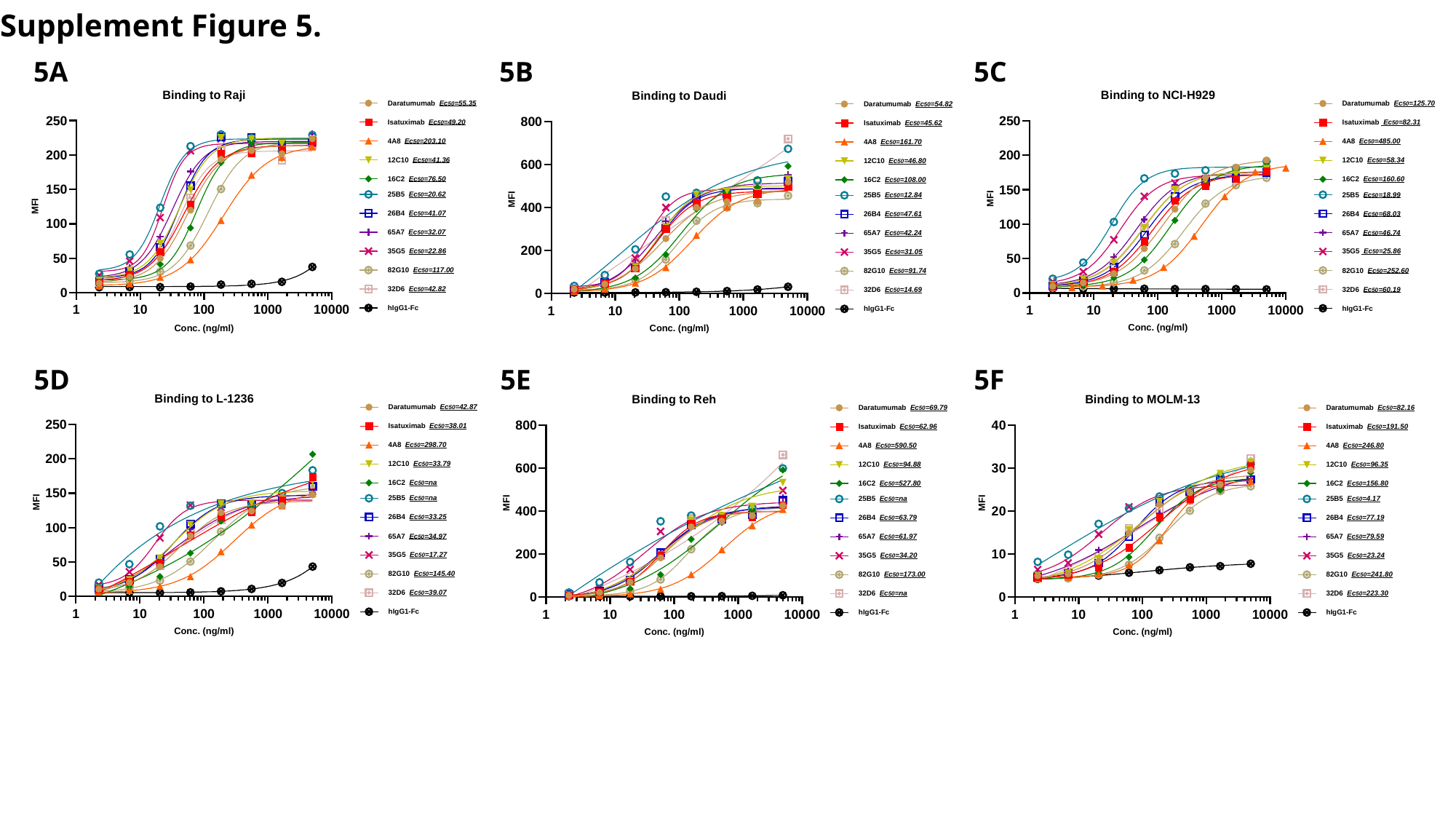

Supplement Figure 5.
5A
5B
5C
5D
5E
5F

## Slide 6
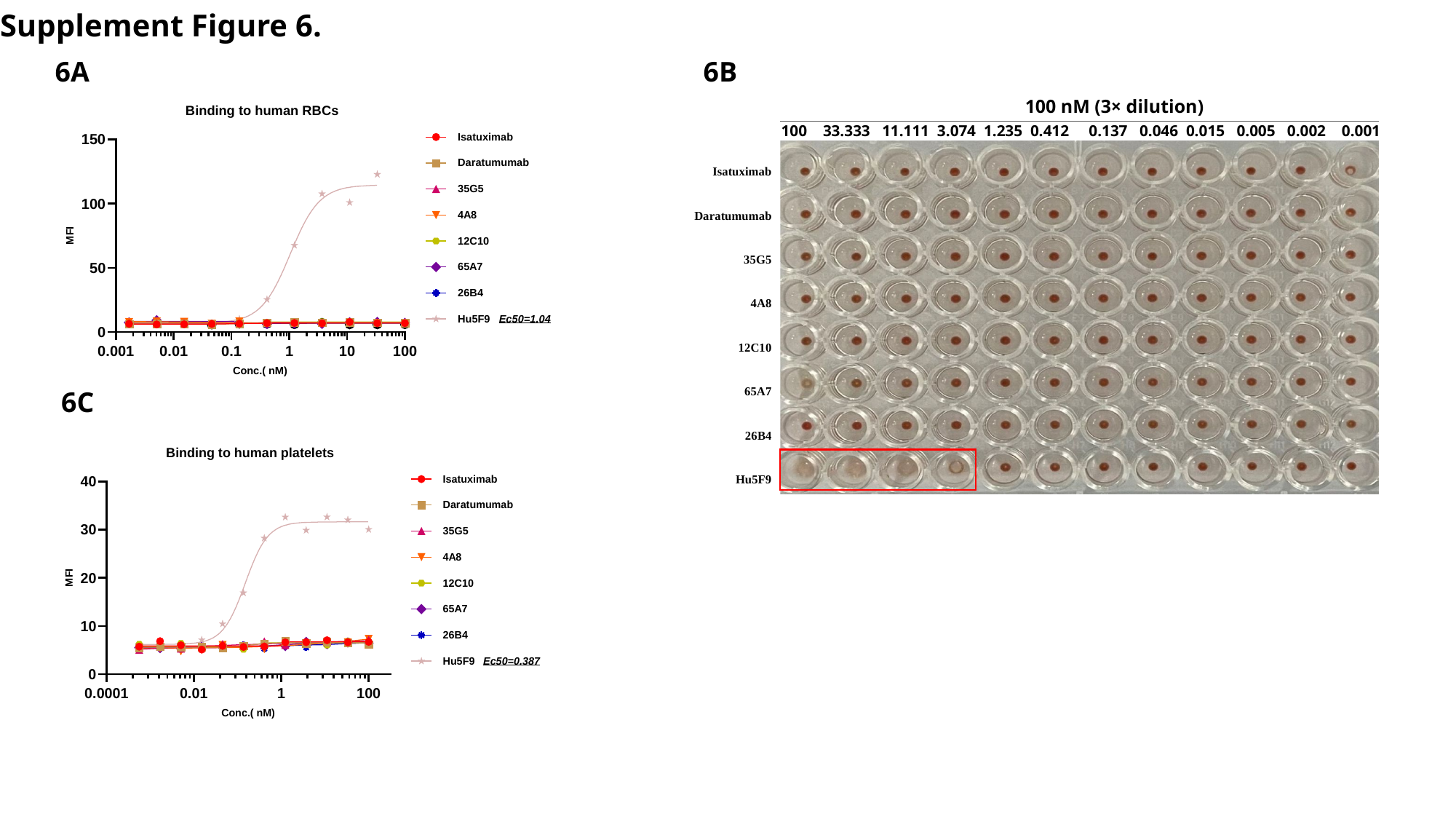

Supplement Figure 6.
6A
6B
100 nM (3× dilution)
100 33.333 11.111 3.074 1.235 0.412 0.137 0.046 0.015 0.005 0.002 0.001
Isatuximab
Daratumumab
35G5
4A8
12C10
65A7
26B4
Hu5F9
6C

## Slide 7
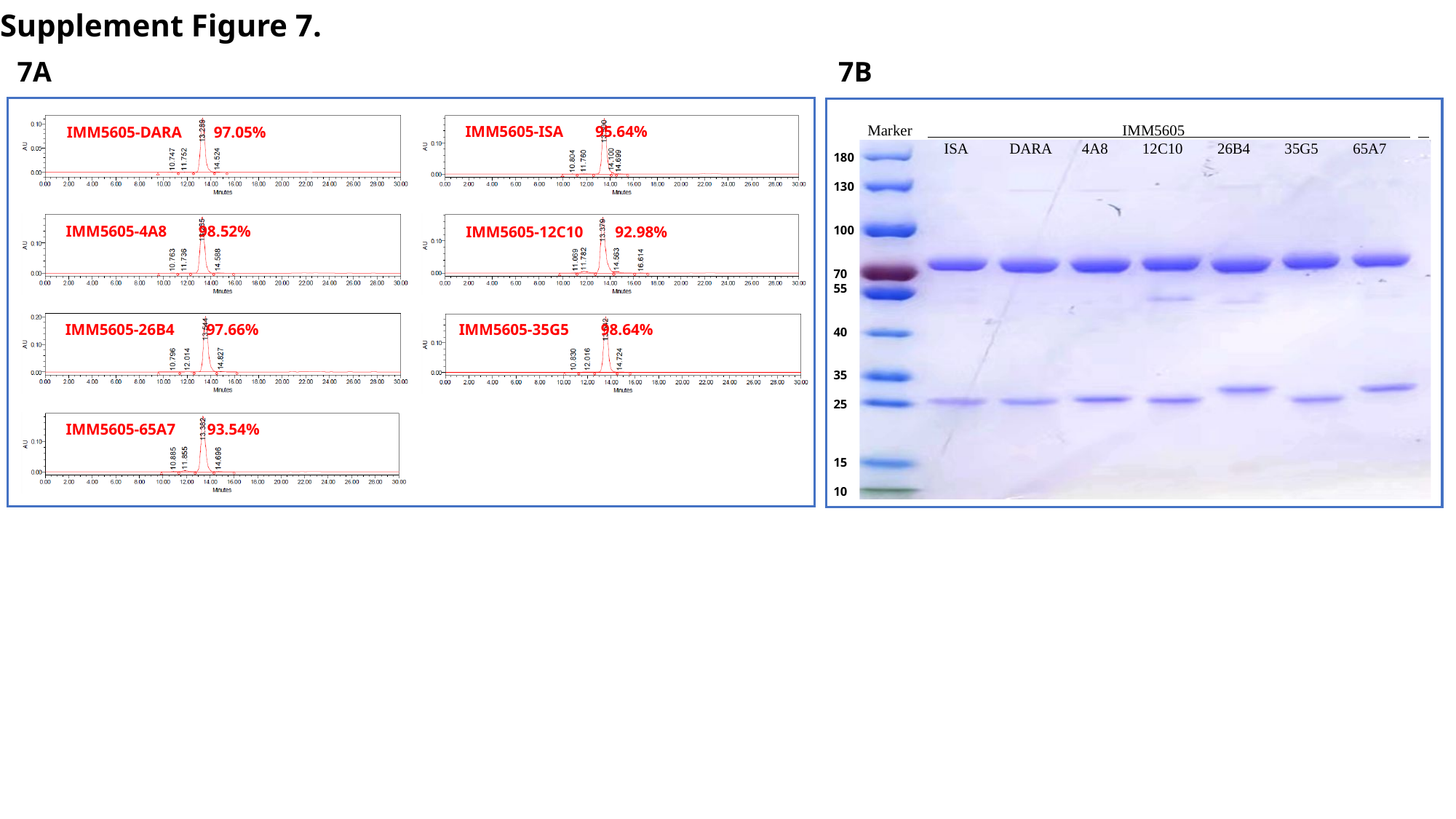

Supplement Figure 7.
7A
7B
IMM5605-ISA 95.64%
IMM5605-DARA 97.05%
IMM5605-4A8 98.52%
IMM5605-12C10 92.98%
IMM5605-26B4 97.66%
IMM5605-35G5 98.64%
IMM5605-65A7 93.54%
Marker IMM5605 1
 ISA DARA 4A8 12C10 26B4 35G5 65A7
180
130
100
70
55
40
35
25
15
10

## Slide 8
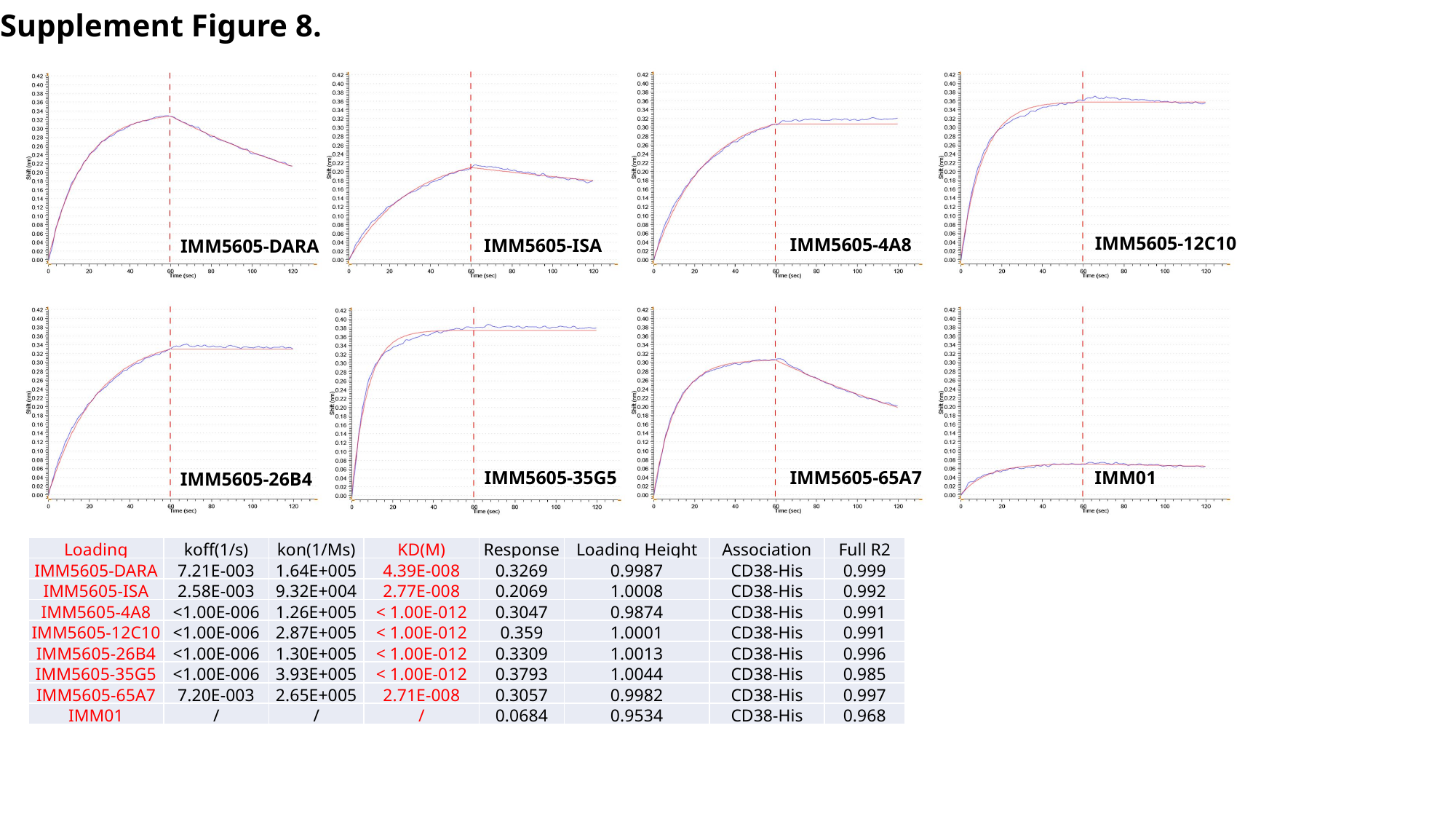

Supplement Figure 8.
IMM5605-12C10
IMM5605-4A8
IMM5605-ISA
IMM5605-DARA
IMM01
IMM5605-65A7
IMM5605-35G5
IMM5605-26B4
| Loading | koff(1/s) | kon(1/Ms) | KD(M) | Response | Loading Height | Association | Full R2 |
| --- | --- | --- | --- | --- | --- | --- | --- |
| IMM5605-DARA | 7.21E-003 | 1.64E+005 | 4.39E-008 | 0.3269 | 0.9987 | CD38-His | 0.999 |
| IMM5605-ISA | 2.58E-003 | 9.32E+004 | 2.77E-008 | 0.2069 | 1.0008 | CD38-His | 0.992 |
| IMM5605-4A8 | <1.00E-006 | 1.26E+005 | < 1.00E-012 | 0.3047 | 0.9874 | CD38-His | 0.991 |
| IMM5605-12C10 | <1.00E-006 | 2.87E+005 | < 1.00E-012 | 0.359 | 1.0001 | CD38-His | 0.991 |
| IMM5605-26B4 | <1.00E-006 | 1.30E+005 | < 1.00E-012 | 0.3309 | 1.0013 | CD38-His | 0.996 |
| IMM5605-35G5 | <1.00E-006 | 3.93E+005 | < 1.00E-012 | 0.3793 | 1.0044 | CD38-His | 0.985 |
| IMM5605-65A7 | 7.20E-003 | 2.65E+005 | 2.71E-008 | 0.3057 | 0.9982 | CD38-His | 0.997 |
| IMM01 | / | / | / | 0.0684 | 0.9534 | CD38-His | 0.968 |

## Slide 9
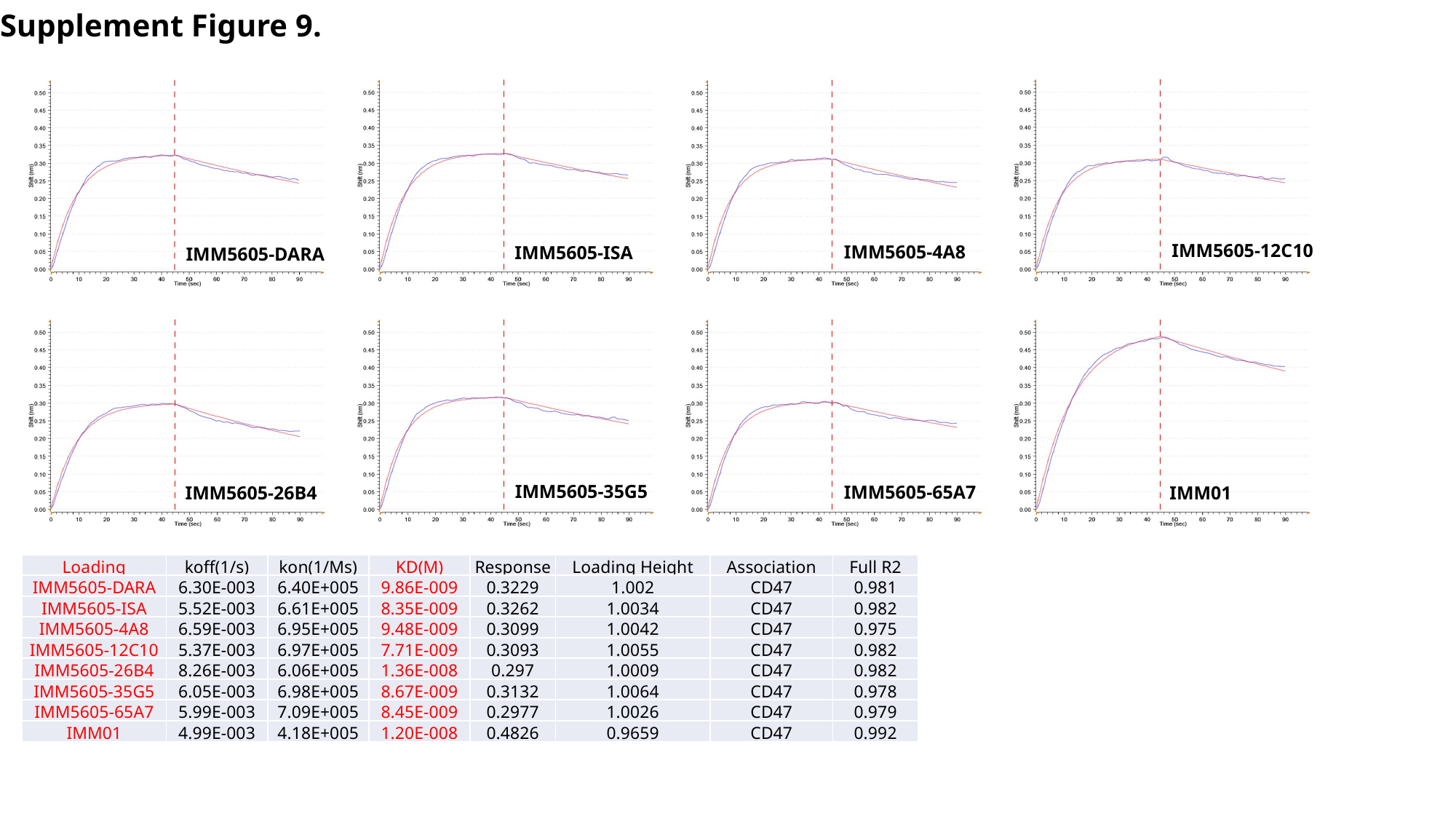

Supplement Figure 9.
IMM5605-12C10
IMM5605-4A8
IMM5605-ISA
IMM5605-DARA
IMM5605-35G5
IMM5605-65A7
IMM5605-26B4
IMM01
| Loading | koff(1/s) | kon(1/Ms) | KD(M) | Response | Loading Height | Association | Full R2 |
| --- | --- | --- | --- | --- | --- | --- | --- |
| IMM5605-DARA | 6.30E-003 | 6.40E+005 | 9.86E-009 | 0.3229 | 1.002 | CD47 | 0.981 |
| IMM5605-ISA | 5.52E-003 | 6.61E+005 | 8.35E-009 | 0.3262 | 1.0034 | CD47 | 0.982 |
| IMM5605-4A8 | 6.59E-003 | 6.95E+005 | 9.48E-009 | 0.3099 | 1.0042 | CD47 | 0.975 |
| IMM5605-12C10 | 5.37E-003 | 6.97E+005 | 7.71E-009 | 0.3093 | 1.0055 | CD47 | 0.982 |
| IMM5605-26B4 | 8.26E-003 | 6.06E+005 | 1.36E-008 | 0.297 | 1.0009 | CD47 | 0.982 |
| IMM5605-35G5 | 6.05E-003 | 6.98E+005 | 8.67E-009 | 0.3132 | 1.0064 | CD47 | 0.978 |
| IMM5605-65A7 | 5.99E-003 | 7.09E+005 | 8.45E-009 | 0.2977 | 1.0026 | CD47 | 0.979 |
| IMM01 | 4.99E-003 | 4.18E+005 | 1.20E-008 | 0.4826 | 0.9659 | CD47 | 0.992 |

## Slide 10
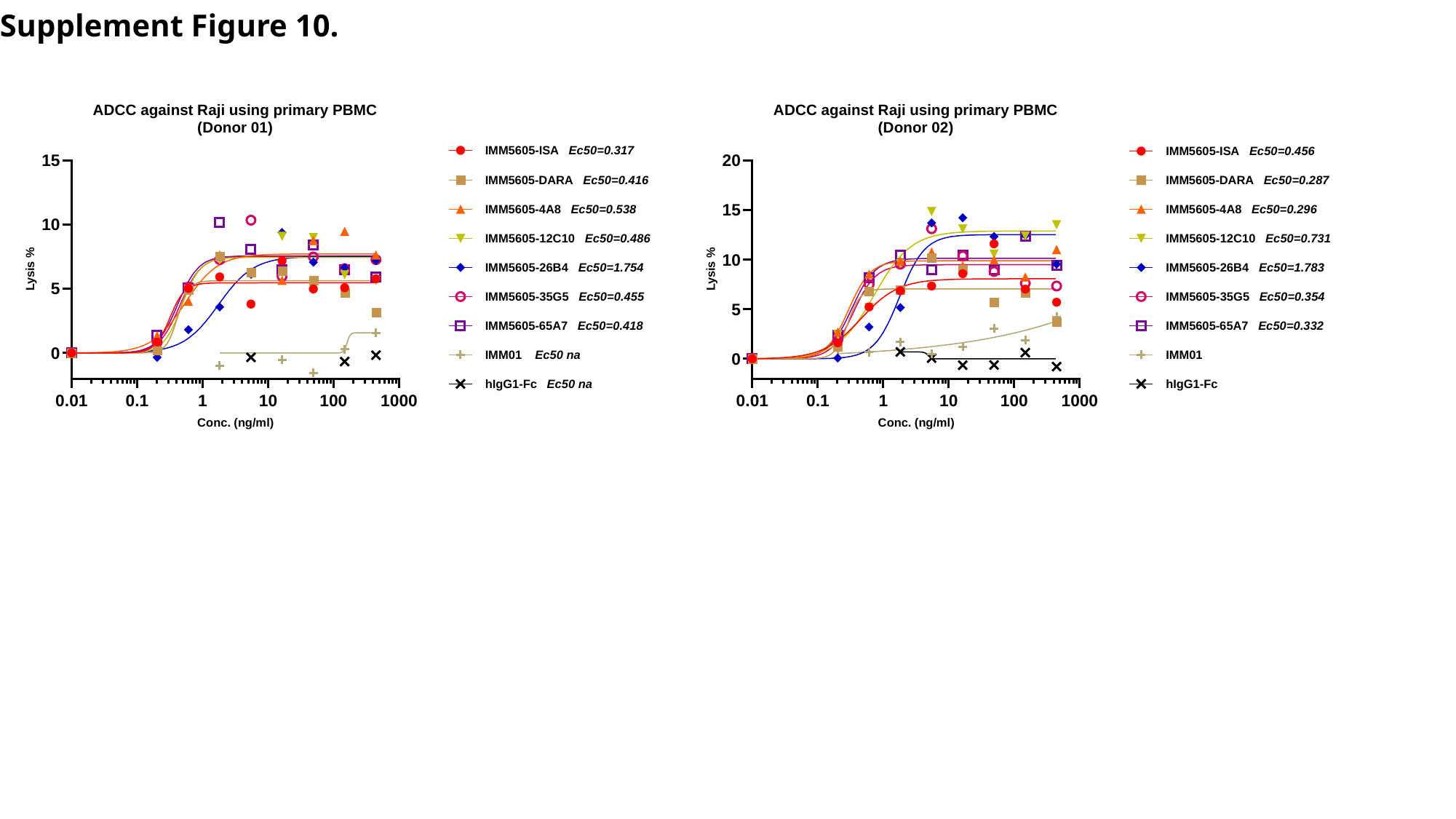

Supplement Figure 10.
